# Screening of potential key ferroptosis-related genes in sepsis

**DOI:** 10.7717/peerj.13983

**Published:** 2022-09-13

**Authors:** Shunan Cui, Kun Niu, Yining Xie, Shuo Li, Wenzhi Zhu, Ling Yu, Hongyu Tan

**Affiliations:** Anesthesiology, Peking University Cancer Hospital & Institute, Beijing, Beijing, China

**Keywords:** Sepsis, Ferroptosis, MAPK, Inflammation, Bioinformatics analysis

## Abstract

**Background:**

Sepsis leads to multiple organ dysfunction caused by a dysregulated host response to infection with a high incidence and mortality. The effect of ferroptosis on the development of sepsis remains unclear. In this study, we aimed to identify the key ferroptosis-related genes involved in sepsis and further explore the potential biological functions of these ferroptosis-related genes in sepsis using bioinformatics analysis.

**Methods:**

The GSE13904 (from children) and GSE28750 (from adults) datasets were downloaded from the Gene Expression Omnibus (GEO). The ferroptosis-related genes were obtained from the FerrDb database. The ferroptosis-related differentially expressed genes (DEGs) were screened by the limma R package. The DAVID online database or clusterProfiler R package was used for the functional enrichment analysis. Then, the STRING database was used to predict the interactions of proteins, and the CytoHubba plugin of Cytoscape was used to confirm key clustering modules. Then, the miRNAs and lncRNAs associated with the key clustering modules were predicted by miRWalk 2.0 and LncBase v.2 respectively. Finally, we generated a cecal ligation and puncture (CLP) polymicrobial sepsis model in C57 male mice and examined the expression of the mRNAs and noncoding RNAs of interest in peripheral blood leukocytes by PCR during the acute inflammation phase.

**Results:**

In total, 34 ferroptosis-related DEGs were identified in both adult and pediatric septic patients. These ferroptosis-related DEGs were mainly enriched in inflammatory pathways. Then, a significant clustering module containing eight genes was identified. Among them, the following five genes were closely associated with the MAPK signaling pathway: MAPK14, MAPK8, DUSP1, MAP3K5 and MAPK1. Then, crucial miRNAs and lncRNAs associated with biomarker MAPK-related genes were also identified. In particular, let-7b-5p and NEAT1 were selected as noncoding RNAs of interest because of their correlation with ferroptosis in previous studies. Finally, we examined the mRNAs, miRNAs and lncRNAs of interest using CLP-induced sepsis in peripheral blood leukocytes of mice. The results showed that MAPK14, MAPK8, MAP3K5, MAPK1 and NEAT1 were upregulated, while DUSP1 and let-7b-5p were downregulated in the CLP group compared with the sham group.

**Conclusions:**

The MAPK signaling pathway may play a key role in regulating ferroptosis during sepsis. This study provides a valuable resource for future studies investigating the mechanism of MAPK-related ferroptosis in sepsis.

## Introduction

Sepsis is defined as a systemic inflammatory response syndrome (SIRS) that occurs when a dysregulated host response to bacterial, viral or fungal infection leads to unacceptably severe tissue damage and organ dysfunction (Keeley, Hine & Nsutebu, 2017). More specifically, sepsis can be viewed as a fierce competition between pathogens and the host immune response, in which pathogens impair all aspects of host immune defense (Hotchkiss, Monneret & Payen, 2013). Once the host loses control of local infection, the activated innate immune system induces a state of robust inflammation characterized by an exorbitant release of inflammatory cytokines, which is also known as a “cytokine storm” and plays an important role in initiating organ dysfunction (Delano & Ward, 2016). Although some septic patients survive this hyperinflammatory phase, they are still at an increased risk for the development of secondary persistent inflammation due to sepsis-induced long-term immunosuppression (Delano & Ward, 2016; Nolt et al., 2018). This immunosuppression is characterized by the deprivation of delayed hypersensitivity to positive control antigens, a failure to eliminate the primary infection and the development of new opportunistic infections (Hotchkiss et al., 2009). With the deepening of research, the extensive loss of immune cells has been considered a major manifestation of sepsis-induced immunosuppression, which is characterized by programmed cell death induction (Cao, Yu & Chai, 2019). Over the past decade, a newly discovered necrotic-form of programmed cell death named ferroptosis has been proposed. The latest study reported that ferroptosis could release danger-associated molecular patterns (DAMPs) that irreversibly trigger stress-exposed cells into a proinflammatory state (Liu et al., 2022). Thus, we hypothesized that ferroptosis may play important roles in the development of inflammation. However, the mechanism of ferroptosis in sepsis-induced systemic inflammation remains unclear.

Ferroptosis is a novel type of cell death characterized by increased lipid reactive oxygen species (ROS), iron-dependent lipid peroxidation and plasma membrane damage (Dixon et al., 2012). Ferroptosis is well known for its specific physiological and morphological characteristics such as iron accumulation, decreased cystine uptake, intact nuclei and shrunken mitochondria, which are different from the characteristics of apoptosis and pyroptosis (Han et al., 2020). In accordance with its name, intracellular iron homeostasis plays a significant role in regulating ferroptosis. Iron metabolism-related genes, such as transferring (TF), transferrin receptor (TFR) and ferroportin (FPN), are considered key mediators of ferroptosis (Mou et al., 2019). For example, the overexpression of heat shock protein beta-1 (HSPB1) inhibits ferroptosis by reducing intracellular iron accumulation by inhibiting TRF1 expression in tumor cells (Li et al., 2020a). Although HSPB1 has been widely considered as a molecular chaperone, upon phosphorylation, HSPB1 dissociates into monomers that act as negative regulators of ferroptosis through reducing the uptake of cellular iron and the production of lipid ROS (Sun et al., 2015). In addition, excessive redox active divalent iron (Fe^2+^) can provide electrons to hyperoxide to form harmful ROS triggering ferroptosis (Battaglia et al., 2020). It has been confirmed that the activation of Ras-mitogen-activated protein kinase (MEK) signaling sensitizes tumor cell lines to ferroptosis by promoting ROS accumulation by inhibiting cystine and mitochondrial voltage-dependent anion channel 2/3 (VDAC 2/3) (Dixon et al., 2012; Han et al., 2020). Despite the evidence emphasizing the significant roles of ferroptosis in cancer, few studies have focused on its function in other diseases. Recently, the roles of ferroptosis in inflammation-related diseases have attracted widespread attention due to its strong association with immunogenicity (Sun et al., 2020). The latest studies reported that the pharmacological suppression of ferroptosis protects against sepsis-induced cardiac injury and liver injury in mice (Li et al., 2020b; Wei et al., 2020). Furthermore, Gong et al. (2022) have identified ferroptosis-related biomarkers of septic cardiomyopathy *via* bioinformatics analysis. Thus, we have reason to believe that ferroptosis plays key roles in sepsis-induced multiple organ injury. However, the specific mechanism and genes associated with ferroptosis in sepsis, especially in sepsis-induced systemic inflammation, are still unknown. It is necessary to further understand the biological functions of ferroptosis-related genes in sepsis.

Here, we first investigated ferroptosis-related differentially expressed genes (DEGs) in public datasets of adult and pediatric septic patients. Then, a functional analysis of ferroptosis-related DEGs and an analysis of the relationships among proteins were performed to understand the biological mechanisms. Furthermore, we explored crucial microRNAs (miRNAs) and long noncoding RNAs (lncRNAs) associated with biomarker genes. Finally, we examined the screened mRNAs, miRNAs and lncRNAs of interest using cecal ligation and puncture (CLP)-induced sepsis in mice. Our results may provide useful information regarding the mechanism of ferroptosis in sepsis, which could help us discover new targets for the clinical diagnosis of sepsis.

## Materials and Methods

### Data collection

We selected the GSE13904 and GSE28750 expression profiling datasets, which were downloaded from the Gene Expression Omnibus (GEO) databases (http://www.ncbi.nlm.nih.gov/geo/). GSE13904 includes 52 whole blood samples from septic children and 18 control samples. GSE28750 includes 10 whole blood samples from septic adults and 20 samples from healthy adults. Both datasets were collected using the same supplementary microarray probe platform GPL570 (Affymetrix Human Genome U133 Plus 2.0 Array). In addition, ferroptosis-related genes were obtained from the FerrDb database (http://zhounan.org/ferrdb/current/) according to previous studies (Hassannia, Vandenabeele & Vanden Berghe, 2019; Yifan, Jianfeng & Jun, 2021). All 247 human expressed ferroptosis-related genes are listed in Table S1.

### Differential ferroptosis-related gene expression

First, the expression profiles of ferroptosis-related genes were determined in each sample from the septic patient and control groups. The ferroptosis-related DEGs were identified using the limma R package (version 3.42.2) based on *|log2 (fold-change)| >* 0.5 and *adjusted p value* < 0.05 (Ritchie et al., 2015). Then, the ggplot2 R package (version 3.3.3) was used to visualize the DEGs by mapping volcano plots of GSE13904 and GSE28750. Moreover, a DEG-related heatmap was generated by the complex-heatmap R package (version 2.2.0). Finally, the Venny 2.1 tool was used to generate a Venn diagram to visualize the DEGs coexpressed in the two datasets.

### Functional enrichment analysis

The DAVID online database or clusterProfiler R package (version 3.14.3) was used for the Gene Ontology (GO) analysis (including biological process (BP), cell composition (CC), and molecular function (MF)) and Kyoto Encyclopedia of Genes and Genomes (KEGG) pathway enrichment analysis (da Huang, Sherman & Lempicki, 2009). Moreover, the Metascape tool was used to analyze the biological pathways (Zhou et al., 2019). The results with *p* < 0.05 were considered to be statistically significant and were visualized with histograms, chord plots, bubble charts or network diagrams.

### Protein–protein interaction (PPI) networks

The STRING database was used to predict the interactions of proteins (Szklarczyk et al., 2019). Interactions with medium confidence (confidence score ≥ 0.4) between two proteins were retained. Cytoscape (version 3.8.2) was used to construct the PPI network (Shannon et al., 2003). Additionally, the key clustering modules were confirmed by the CytoHubba plugin of Cytoscape, with a maximal clique centrality (MCC) score >4 (Chin et al., 2014). Finally, GO and KEGG enrichment analyses of the key clustering modules were performed (Yu et al., 2012), and *p* < 0.05 was considered the threshold.

### Analysis of mRNA–miRNA and miRNA–lncRNA interactions

miRWalk 2.0 was used to predict miRNAs associated with the key clustering modules (Dweep et al., 2011). To ensure that all predicted miRNAs were verified in previous experiments, both the miRWalk and miRTarBase databases were used to confirm specific gene-related miRNAs. Furthermore, miRNAs targeting more than two genes were selected for the subsequent prediction of upstream lncRNAs. LncBase v.2 was used to identify lncRNAs (Paraskevopoulou et al., 2016). Finally, mRNA**–**miRNA and mRNA**–**miRNA-lncRNA interaction network diagrams were generated by Cytoscape.

### Ethical statements and CLP-induced sepsis model

All animal experiments complied with the Chinese guidelines for experimental animals and were approved by the Animal Ethics Committee of Peking University Cancer Hospital and Institute (certification number for animal use permission: 110324220100257736). Six- to 8-week-old male C57BL/6J mice weighing 22–24 g (Sipeifu, Beijing, China) were housed in a pathogen-free environment under a 12 h light-dark cycle, and adequate food and water were provided. The environmental temperature was maintained at 18–22 °C and the humidity was maintained at 45–55%.

CLP is the most widely used sepsis model because of its similarity with the clinical manifestations of septic patients and its excellent simulation of the pathological process of sepsis (Hubbard et al., 2005). According to our previous study, the CLP model was established (Cui et al., 2019). First, mice were randomly divided into two groups with 10 animals per group using Microsoft Excel (the randomization sequence is listed in the Supplemental File). All mice were labeled with ear tags for the subsequent experiments. Briefly, the mice were anesthetized with isoflurane. Then, we shaved and disinfected the abdomen of the mice using a depilatory agent and 75% alcohol and performed a longitudinal midline skin incision. After cecal exposure, sterile silk was used to ligate the cecum under fixed conditions, and a 20-gauge needle was used to double puncture the cecum to induce mid-grade sepsis. Next, we extruded a fixed amount of feces out of the hole and returned the cecum into the abdomen. In the sham group, the exposed cecum was not ligated or punctured and was then returned to the abdomen. We performed fluid resuscitation by injecting 1 ml 0.9% normal saline subcutaneously into the necks of mice after modeling. Twenty-four hours after the CLP procedure, isoflurane was used to anesthetize the mice. To ensure that the mice lose consciousness and were sacrificed with minimal pain, we euthanized the mice by rapid neck dislocation, and collected their peripheral blood and organs (lung, kidney and liver) for further experiments.

### Real-time quantitative polymerase chain reaction (qRT–PCR) and stem–loop qRT–PCR

Red blood cells in whole blood were lysed with lysis buffer (Absin, Shanghai, China), and the total RNA was isolated from leukocytes. According to a previous study, stem**–**loop reverse transcription and qRT–PCR were used to measure the expression of let-7b-5p (Hong et al., 2020). U6 was used as an internal control. qRT–PCR was used to measure the expression of lncRNAs and mRNAs. Glyceraldehyde 3-phosphate dehydrogenase (GAPDH) was used as an internal control. cDNA was produced according to the RT kit instructions (Takara, Dalian, China). qRT–PCR was carried out by using SYBR-Green (Takara, China). The fold change was calculated using the 2^−ΔΔCT^ method. The PCR primers were synthesized by Tsingke Biological Technology in Beijing, China. The sequences were listed in Table S2.

### Statistical analysis

The data are expressed as the mean ± standard error of the mean (s.e.m.) of six replicate experiments. The qRT–PCR results were analyzed by an investigator blinded to the group using *t tests*. GraphPad Prism 6 for Windows (San Diego, CA, USA) was used for the statistical analysis and drawing the graphics. *P* < 0.05 was considered as significant differences.

## Results

### Identification of ferroptosis-related DEGs

**Figure 1:**
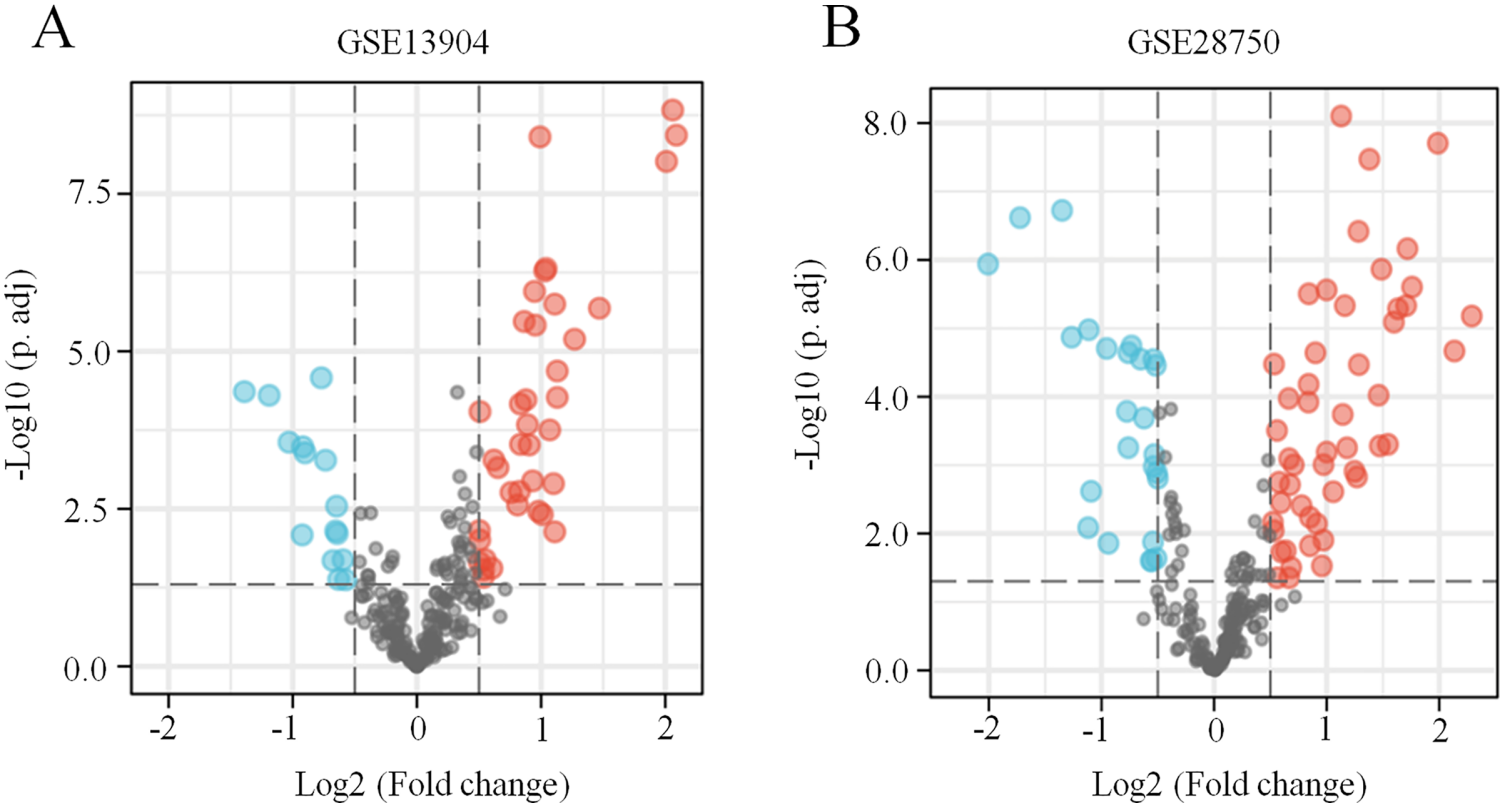
Identification of ferroptosis-related DEGs in sepsis. (A) A volcano plot showing ferroptosis-related DEGs from the blood samples of septic children and controls in the GSE13904 dataset. (B) A volcano plot showing ferroptosis-related DEGs from the blood samples of septic adults and controls in the GSE28750 dataset. The upregulated genes are marked in red, and the downregulated genes are marked in blue. *|log2 (fold-change)| >* 0.5 and *adjusted p* < 0.05 were considered statistically significant. The black points represent no significant difference.

**Figure 2:**
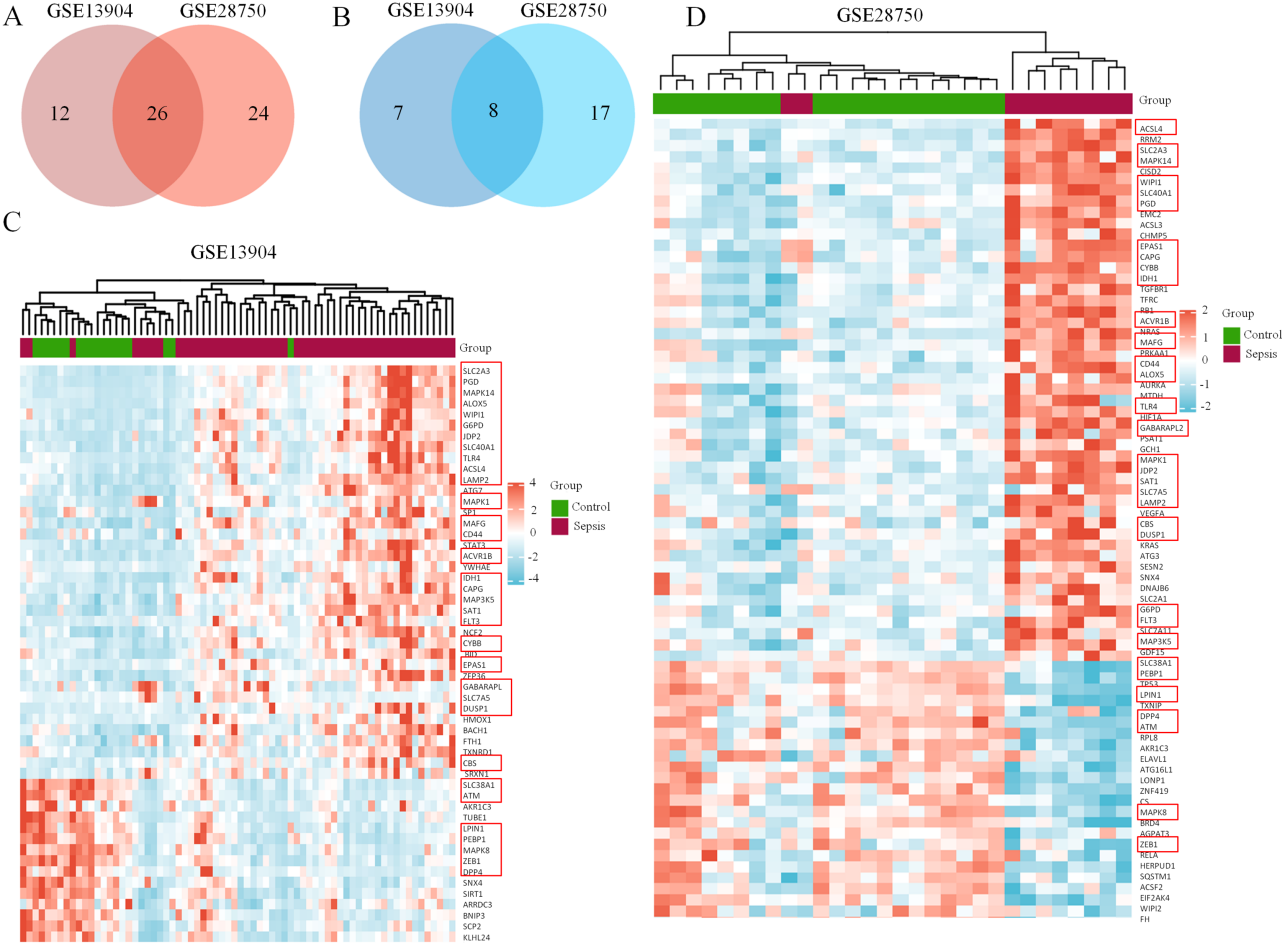
Coexpression network of ferroptosis-related DEGs in septic children and adults. (A) Intersection of upregulated ferroptosis-related DEGs in the GSE13904 and GSE28750 datasets. (B) Intersection of downregulated ferroptosis-related DEGs in the GSE13904 and GSE28750 datasets. The count on the left, ferroptosis-related DEGs unique to GSE13904; the count in the middle, ferroptosis-related DEGs common to both datasets; and the count on the right, ferroptosis-related DEGs refer to GSE28750. The heatmap shows the expression of the ferroptosis-related DEGs from GSE13904 (C) and GSE28750 (D). Red represents significantly upregulated genes and blue represents significantly downregulated genes in the samples. The 34 coexpressed ferroptosis-related DEGs were circled by red rectangles.

First, the GSE13904 (from children) and GSE28750 (from adults) datasets were downloaded from GEO. The Uniform Manifold Approximation and Projection (UMAP) and the principal component analysis (PCA) were used to check and visualize the grouped data. The results of UMAP showed that the gene transcriptional profiles of septic patients and controls were distinct from each other in both datasets (Figs. S1A and S1B). Similarly, the PCA plots showed that the two principal components contained 44.0% of the variance in GSE13904 and 59.1% of the variance in GSE28750 (Figs. S1C and S1D). In addition, the normalized signal intensity for each sample was analyzed in two datasets, respectively. The boxplots showed that the signal intensity of each sample was almost at the same median level (Figs. 1E and 1F). Thus, good normalization and repeatability were exhibited in the samples in these two datasets, which was a prerequisite for the subsequent analyses. Then, the ferroptosis-related DEGs were confirmed with a threshold of *|log2 (fold-change)|* > 0.5 and *adjusted p value* < 0.05. In GSE13904, a total of 53 ferroptosis-related DEGs (38 upregulated and 15 downregulated genes) were identified between the septic children and the control group. In GSE28750, a total of 75 ferroptosis-related DEGs (50 upregulated and 25 downregulated genes) were identified between the septic adults and the control group. The results are shown in volcano plots in Figs. 1A and 1B. The detailed DEG expression files are shown in Tables S3 and S4. In addition, a total of 34 genes including 26 upregulated and 8 downregulated genes showed apparent differences between the septic children and adults compared with those in the control groups in the two datasets (Figs. 2A, 2B and Table 1). And these 34 genes were marked in heatmaps that showed ferroptosis-related DEGs from GSE13904 (Fig. 2C) and GSE28750 (Fig. 2D).

**Table 1:** Summary of 34 ferroptosis-related DEGs in sepsis.

	Driver	Marker	Suppressor
UP	PGD, MAPK14, WIPI1, G6PD, TLR4, ACSL4, MAPK1, ACVR1B, IDH1, SAT1, FLT3, CYBB, EPAS1, GABARAPL2	SLC2A3, ALOX5, JDP2, SLC40A1, MAFG, CAPG, MAP3K5, SLC7A5, DUSP1, CBS	LAMP2, CD44
DOWN	ZEB1, ATM, DPP4, LPIN1, PEBP1, SLC38A1, MAPK8		AKR1C3

### Functional and pathway enrichment analysis of ferroptosis-related DEGs

**Figure 3:**
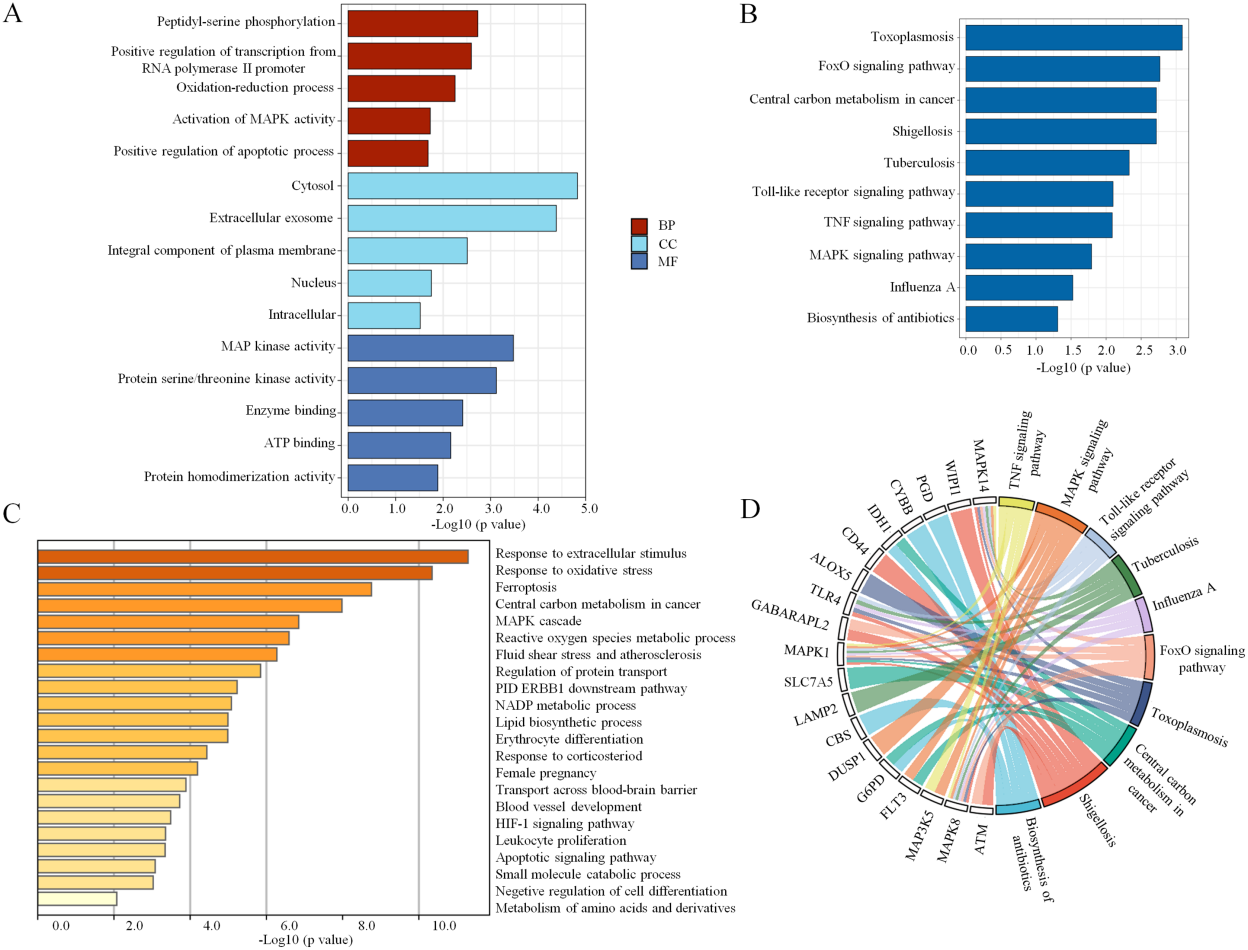
The enrichment of GO terms and KEGG pathways based on ferroptosis-related DEGs in sepsis. (A) GO enrichment analysis of the ferroptosis-related DEGs, including biological process (BP), cell composition (CC), and molecular function (MF). (B) The top 10 most significant KEGG pathways with the largest number of enriched genes are selected and shown. (C) The top 20 biological pathways analyzed by Metascape are shown. (D) A chord plot shows the correlation between the top 10 KEGG pathway terms and their participating genes. A *p* < 0.05 is considered statistically significant.

To explore the pathophysiological functions of the 34 coexpressed ferroptosis-related DEGs, GO and KEGG pathway analyses were performed. We found that peptidyl-serine phosphorylation, oxidation–reduction process, activation of mitogen- activated protein kinase (MAPK) activity and positive regulation of apoptotic process were significantly enriched among BPs. In addition, the statistically significant CC terms mainly included cytosol, extracellular exosome, integral component of plasma membrane, nucleus and intracellular. In the MF analysis, the enriched terms included MAP kinase activity, protein serine/threonine kinase activity, enzyme binding, adenosine triphosphate (ATP) binding and protein homodimerization activity. These results are shown in Fig. 3A. Subsequently, the KEGG pathway analysis revealed that the main enriched pathways were involved in the inflammatory response, including the forkhead box O (FoxO) signaling pathway, Toll-like receptor signaling pathway, tumor necrosis factor (TNF) signaling pathway and MAPK signaling pathway (Fig. 3B). In addition, a chord plot revealed that more than half of the coexpressed ferroptosis-related DEGs (19 of 34) were involved in the regulation of the top ten enriched KEGG pathways (Fig. 3D). Specific information regarding the statistically significant terms (BP, CC, MF and KEGG pathway analyses) with the number of enriched genes are listed in Table S5. Moreover, the biological function enrichment analysis of these 34 ferroptosis-related DEGs by Metascape indicated similar results and revealed the same terms such as central carbon metabolism in cancer, MAPK cascade and apoptotic signaling pathway (Fig. 3C). Overall, the MAPK signaling pathway seemed to be the key factor that modulated ferroptosis in sepsis.

### Identification of key ferroptosis-related genes involved in sepsis

**Figure 4:**
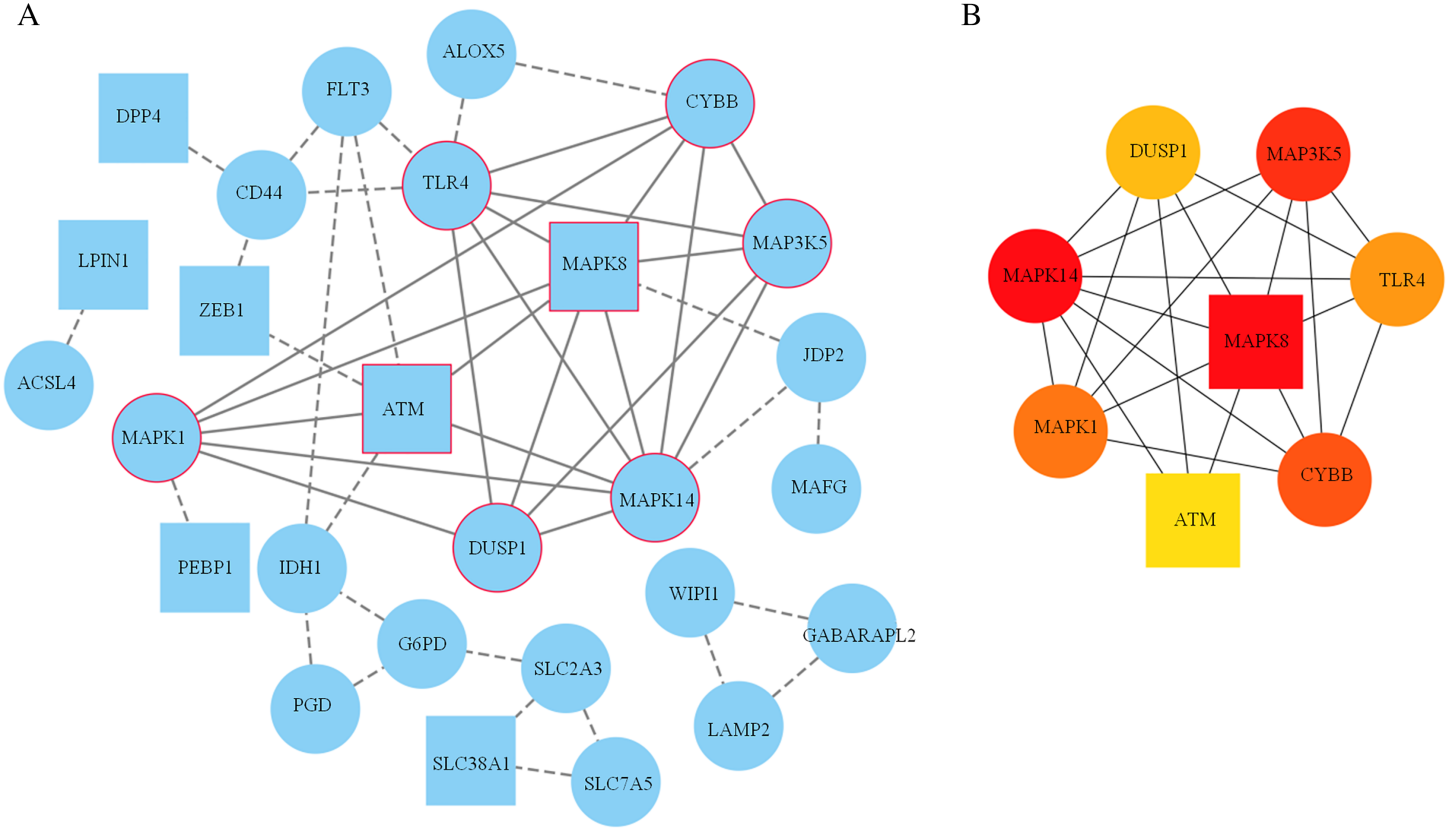
Construction of the PPI network. (A) 27 blue nodes and 47 edges representing genes and interactions between genes respectively were selected and visualized according to an interaction score ≥0.4. According to the MCC score >4, a significant clustering module composed of eight key genes is circled in red. (B) The network of these eight key genes was visualized. The red node represents the highest MCC score, and the yellow node represents the lowest MCC score. Circles represent upregulated genes. Squares represent downregulated genes.

**Figure 5:**
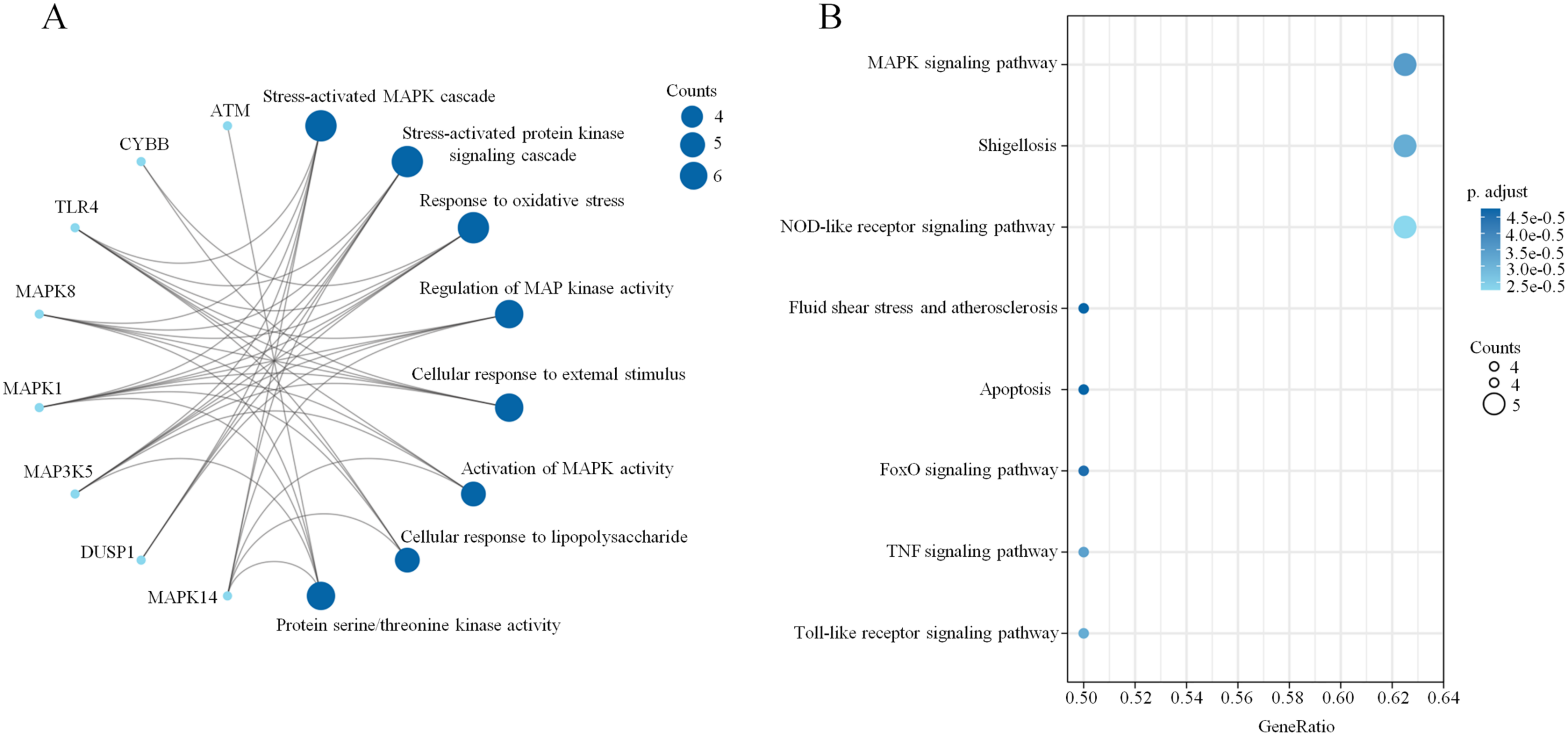
Biological pathway enrichment of the clustering module. (A) A network diagram of the top eight GO terms. (B) A bubble chart of the top eight KEGG pathways. The bubble area is positively proportional to the number of genes in a certain GO term or KEGG pathway. An *adjusted p* < 0.01 was considered statistically significant.

A PPI network based on the 34 ferroptosis-related DEGs was constructed and visualized by selecting the proteins with an interaction score ≥0.4 (Fig. 4A). The results indicated that there were 27 nodes and 47 edges that represented genes and interactions between genes respectively (Table S6). Meanwhile, a significant clustering module composed of eight key genes which included six upregulated genes (MAPK14, MAPK1, MAP3K5, toll-like receptor (TLR) 4, cytochrome B-245 beta chain (CYBB) and dual specificity phosphatase (DUSP) 1) and two downregulated genes (MAPK8 and ataxia telangiectasia-mutated (ATM)) was screened with a threshold of MCC score >4 (Fig. 4B). The MCC scores of the 27 interacting proteins are listed in Table S7. Subsequently, a biological pathway enrichment analysis was performed to identify the functions of these eight key genes. The GO and KEGG pathway results, showed that the MAPK signaling pathway was significantly enriched (*adjusted p value* = 3.53E−05). The top eight GO terms and KEGG pathways are illustrated in a network diagram and a bubble chart, respectively (Figs. 5A and 5B). Moreover, five of the eight identified genes (MAPK14, MAPK8, DUSP1, MAP3K5 and MAPK1) were involved in the MAPK signaling pathway, and the specific enrichment information is listed in Table S8.

### Construction of the mRNA–miRNA–lncRNA network

**Figure 6:**
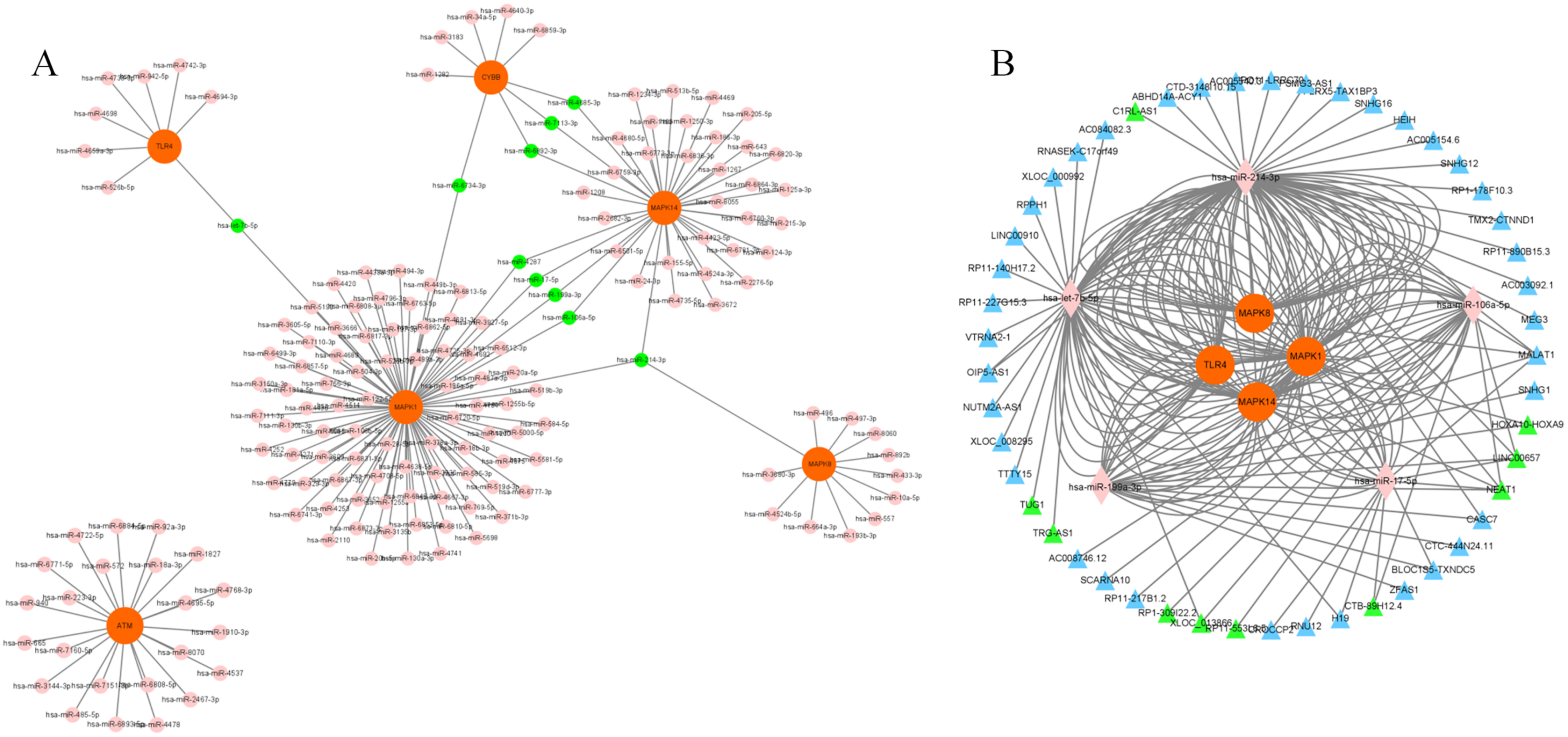
Construction of mRNA–miRNA-lncRNA network. (A) Interaction of key genes with their targeted miRNAs. A total of 168 miRNAs were identified. *P* < 0.05 was considered statistically significant. Specifically, 10 miRNAs are marked green because they linked several genes. (B) 49 lncRNAs were correlated with 5 miRNAs targeting four genes. The mRNA–miRNA-lncRNA network is shown. Specifically, 10 lncRNAs are marked green according to their predicted score >0.9. mRNAs are colored orange. miRNAs are colored pink. LncRNAs are colored blue.

We chose a significant clustering module composed of eight key genes as genes of interest and analyzed the mRNA–miRNA and miRNA–lncRNA interactions. We identified a total of 168 miRNAs targeting six genes (MAPK14, TLR4, CYBB, MAPK1, MAPK8 and ATM) *via* utilizing both the miRWalk and miRTarBase databases with thresholds of *p* < 0.05 and the 3′UTR as the gene-binding region (Fig. 6A, Table S9). Specifically, 10 miRNAs (hsa-miR-7113-3p, hsa-miR-6892-3p, hsa-miR-4685-3p, hsa-miR-4287, hsa-miR-199a-3p, hsa-miR-17-5p, hsa-miR-106a-5p, hsa-let-7b-5p, hsa-miR-6734-3p and hsa-miR-214-3p) were listed because they linked several genes (≥2, Table 2). LncBase was used to predict the upstream lncRNAs of these 10 miRNAs. Forty-nine lncRNAs were identified to correlate with five miRNAs (hsa-miR-199a-3p, hsa-miR-17-5p, hsa-miR-106a-5p, hsa-let-7b-5p and hsa-miR-214-3p) targeting four genes (MAPK14, TLR4, MAPK1 and MAPK8). The mRNA–miRNA–lncRNA network is shown in Fig. 6B. In addition, among these 49 lncRNAs, 10 lncRNAs (CTB-89H12.4, LINC00657, CTB-89H12.4, RP11-553L6.5, NEAT1, XLOC_013866, RP1-309I22.2, TRG-AS1, TUG1 and HOXA10-HOXA9) had the highest predicted scores (>0.9), illustrating their strong correlation with the targeting miRNAs (Table S10). Previous studies have shown that let-7b-5p and nuclear-enriched abundant transcript 1 (NEAT1) are involved in the regulation of MAPK1 and participate in ferroptosis in cancer cells (Chen, Xin & Zhang, 2019; Du et al., 2019; Dong et al., 2021; Wu & Liu, 2021). However, their roles in sepsis are still unclear. Here, we chose let-7b-5p and NEAT1 for further investigation.

**Table 2:** Summary of 10 miRNAs linked several genes.

miRNA	Target genes		
hsa-miR-7113-3p	CYBB	MAPK14	
hsa-miR-6892-3p			
hsa-miR-4685-3p			
hsa-miR-4287	MAPK1	MAPK14	
hsa-miR-199a-3p			
hsa-miR-17-5p			
hsa-miR-106a-5p			
hsa-let-7b-5p	MAPK1	TLR4	
hsa-miR-6734-3p	CYBB	MAPK1	
hsa-miR-214-3p	MAPK1	MAPK14	MAPK8

### Expression levels of ferroptosis-related mRNAs, miRNAs and lncRNAs in septic mice during acute inflammation

**Figure 7:**
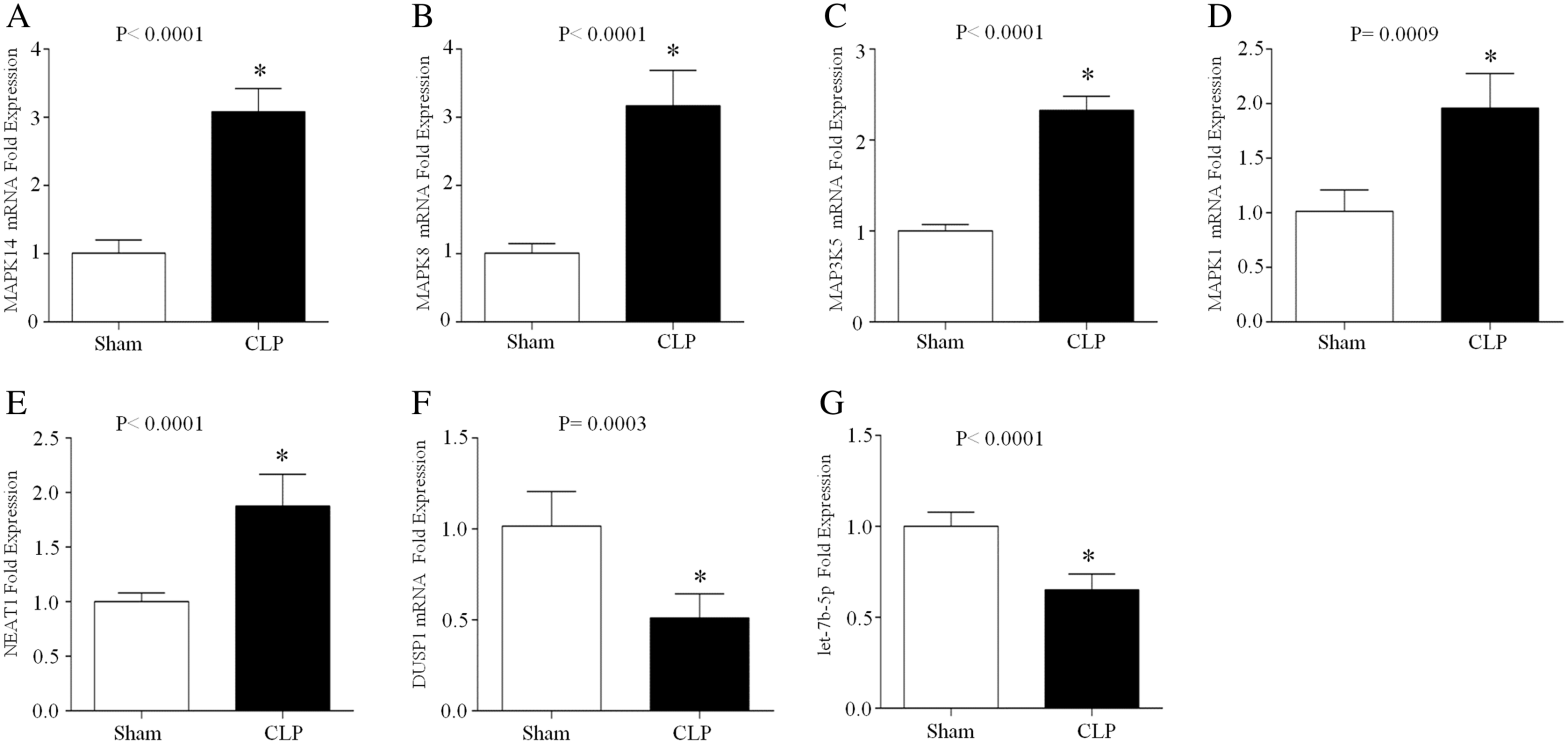
The expression of NEAT1, let-7b-5p and five ferroptosis-related mRNAs in the peripheral blood leukocytes of septic mice. 24 h after the CLP procedure, the peripheral blood leukocytes of mice were collected and total RNA was isolated. The expression of MAPK14 (A), MAPK8 (B), MAP3K5 (C), MAPK1 (D), NEAT1 (E), DUSP1 (F) and let-7b-5p (G) was analyzed by RT–qPCR array. The data represent the mean ± s.e.m of six replicate experiments *P < 0.05.

CLP, which is a model of polymicrobial sepsis, was used to induce acute inflammation in mice. Twenty-four hours after the CLP procedure, 7 of 10 mice survived, and multiorgan (including lung, kidney and liver) injury was observed in six mice, indicating the successful establishment of the sepsis model (Fig. S2). Then, peripheral blood leukocytes were obtained from the CLP group and sham group and the total RNA was extracted. The expression levels of let-7b-5p, NEAT1 and five ferroptosis-related genes (MAPK14, MAPK8, DUSP1, MAP3K5 and MAPK1) were detected by qRT–PCR. The results showed that MAPK14, MAPK8, MAP3K5, MAPK1 and NEAT1 were upregulated in the CLP group compared with those in the sham group, while DUSP1 and let-7b-5p were downregulated in the septic mice during acute inflammation (Fig. 7).

## Discussion

Sepsis is currently regarded as profound systemic inflammation and a dysregulated immune response to invasive pathogens that results in multiple organ injury (Hotchkiss et al., 2016). Notably, disorders of innate and adaptive immunity are important factors that promote the development of sepsis (Hotchkiss et al., 2016). In recent years, a novel form of programmed cell death called ferroptosis has been defined. The immunological consequences of ferroptosis include the death of leukocyte subsets and the corresponding loss of immune function (Tang et al., 2021). Although the specific physiological function of ferroptosis has not been clearly elucidated, emerging evidence demonstrates that ferroptosis is associated with multiple human diseases, such as cancer, degenerative diseases and inflammation-related diseases (Han et al., 2020; Sun et al., 2020). In this study, we analyzed the differences in ferroptosis-related genes between septic patients and healthy controls. Importantly, 26 upregulated and 8 downregulated genes were identified in both septic children and adults. The MAPK signaling pathway seemed to be a key factor in modulating ferroptosis and was associated with these genes in sepsis. Moreover, the significantly differentially expressed noncoding RNAs let-7b-5p and NEAT1, which are involved in MAPK1 regulation, were identified in peripheral blood leukocytes from septic mice. This study may help us better understand the role of ferroptosis in sepsis.

It is essential to identify disease-associated gene sets by coexpression analysis to obtain new insights into disease biology (Li et al., 2021). Therefore, the 34 ferroptosis-related DEGs that were coexpressed in children and adult septic patients were screened out followed by a subsequent enrichment analysis. The GO analysis based on the DAVID database revealed that the oxidation–reduction process, activation of MAPK activity, positive regulation of apoptotic processes and extracellular exosomes were significantly enriched. Regarding cellular reductive/oxidative (redox)-based metabolic processes, imbalanced redox homeostasis drives ferroptosis by causing lethal lipid peroxidation (Jiang, Stockwell & Conrad, 2021). We noticed that the dysregulation of ferrous iron (Fe^2+^) was another important factor leading to ferroptosis. A prior study proved that prominin 2 participates in ferroptosis resistance in mammary epithelial and breast carcinoma cells by promoting exosome-mediated iron export (Brown et al., 2019). Moreover, adenosine 5′-monophosphate (AMP)-activated protein kinase (AMPK)-mediated phosphorylation of acetyl-CoA carboxylase (ACC) and polyunsaturated fatty acid biosynthesis have been confirmed to be involved in ferroptosis (Lee et al., 2020). Although ferroptosis is an iron-dependent form of nonapoptotic regulated cell death, PERK-eIF2α-ATF4-CHOP signaling pathway-mediated p53 upregulated modulator of apoptosis (PUMA) expression is involved in the synergistic interaction between ferroptosis and apoptosis (Lee et al., 2018). Therefore, combined with previous studies, the biological function enrichment results imply that ferroptosis plays a potential role in the development of sepsis. Prior studies have noted a close correlation between ferroptosis and inflammation, as ferroptotic cells produce excessive reactive oxygen species (ROS) (Han et al., 2020). We noticed that both the KEGG pathway analysis and Metascape GO analysis revealed significant enrichment of multiple inflammatory pathways and functions such as the Toll-like receptor (TLR) signaling pathway, tumor necrosis factor (TNF) signaling pathway, MAPK signaling pathway, response to oxidative stress and ROS metabolic process. It is well known that activated TLRs are responsible for the early activation of inflammatory genes and are involved in sepsis-associated cytokine storms (Hotchkiss et al., 2016). TLRs are also involved in iron metabolism in sepsis by transcriptionally downregulating ferroportin (FRP), the exporter of intracellular iron, indicating their roles in ferroptosis (Liu et al., 2021). The TNF signaling pathway and MAPK signaling pathway play important roles in oxidative stress, inflammatory cytokines and programmed cell death. A previous study demonstrated that TNF-α induced the generation of ROS and that extracellular vesicle (EV) release required the upregulation of glutaminase to participate in neuroinflammation (Wang et al., 2017). Because glutaminase is involved in ferroptosis, the TNF signaling pathway may participate in ferroptosis during the inflammatory response. In addition, a recent study focusing on neonatal mice reported that a member of the lipocalin family named lipocalin 2 plays important roles in lipopolysaccharide (LPS)-induced inflammation and oxidative stress by modulating ferroptosis *via* the MAPK/ERK pathway (Wang et al., 2022). Therefore, we have reason to believe that the MAPK signaling pathway may play potential roles in sepsis-induced ferroptosis.

To further reveal the mechanism underlying the biological processes, a PPI analysis based on the 34 ferroptosis-related DEGs was performed. A key clustering module was screened based on the CytoHubba plug-in unit in Cytoscape. The results indicated that 8 genes (MAPK14, MAPK8, TLR4, MAP3K5, CYBB, DUSP1, MAPK1 and ATM) might be closely related to the occurrence of sepsis. Among them, MAPK14, TLR4, MAPK1 and MAPK8 have been confirmed to be involved in proinflammatory cytokine responses, especially in the TLR/IL-1R pathway cascade. A previous study investigating the hyperinflammatory phase during sepsis suggested that activated autophagy degenerated an E3 ubiquitin ligase and scaffold protein called Pellino 3 in TLR4-signaling, resulting in a reduction in phosphorylated MAPK14, MAPK8 and MAPK1 and ultimately inhibiting proinflammatory IL-1β expression (Giegerich et al., 2014). Moreover, CYBB, also known as nicotinamide adenine dinucleotide phosphate (NADPH) oxidase 2 (NOX2), is not only involved in the generation of the autophagy protein LC3 during TLR signaling, but also activated by the Hippo pathway effector PDZ-binding motif (TAZ) to promote ferroptosis (Huang et al., 2009; Yang et al., 2020). Since autophagy regulates cellular iron homeostasis and is required for ferroptosis-associated ROS accumulation, the potential roles of the autophagy-related signaling pathway in ferroptosis have been suggested (Gao et al., 2016). MAP3K5, which is also known as apoptosis signal-regulating kinase 1 (ASK1), has been proven to be associated with erastin- and RAS-selective lethal 3 (RSL3)-induced ferroptosis through the p38/c-Jun N-terminal kinase (JNK) pathway in multiple cell lines (Hattori et al., 2017). A recent study also indicated that ASK1 expression was increased in septic mice and that the inhibition of ASK1 impaired JNK-mediated cytokine production during LPS-induced endothelial inflammation (Miller et al., 2021). In the p38/JUN pathway, a type of phosphatase with dual specificity for tyrosine and threonine named DUSP1 plays an important role in autophagy-related ferroptosis (Chen et al., 2021). Moreover, the ectopic expression of DUSP1 in LPS-stimulated macrophages inhibited the release of TNF-α and IL-6 (Hoppstädter & Ammit, 2019). In contrast, the depletion of DUSP1 resulted in the excessive release of inflammatory cytokines (Hoppstädter & Ammit, 2019). ROS-mediated DNA damage (DDR) is a key factor in ferroptosis. ATM is an important coordinator of the DDR, mainly because the depletion of ATM results in the accumulation of ROS and damage to cellular DNA (Huff, Yan & Clemens, 2021). A previous study indicated that ATM is essential for Ikβ phosphorylation and the translocation of nuclear factor kappa-B (NF-κB) to the nucleus and that ATM participates in the TLR-mediated inflammatory response (Huff, Yan & Clemens, 2021). Importantly, the NF-κB signaling pathway has been verified to be involved in erastin-induced ferroptosis (Oh et al., 2019). These results suggested that changes in MAPK14, TLR4, MAP3K5, CYBB, DUSP1, MAPK1, MAPK8 and ATM may be partially associated with ferroptosis in the sepsis-induced inflammatory response. Next, GO and KEGG analyses of these eight key genes were performed. The results implied that the MAPK signaling pathway, which consists of MAPK14, DUSP1, MAP3K5, MAPK1 and MAPK8 was significantly enriched. A previous study demonstrated that the excessive activation of the MAPK pathway might promote iron-related ROS generation by inhibiting cystine (Cys2) or mitochondrial VDAC2/3 and subsequently induce cell ferroptosis (Han et al., 2020). In addition to ROS generation, MAPK is associated with iron metabolism-related inflammatory signaling. For example, the administration of ferritin light chain (FTL) to macrophages suppressed ERK1/2 activation, decreased the production of NF-κB-induced proinflammatory factors and protected septic mice (Zarjou et al., 2019). The increase in glutathione peroxidase (GPX) 4 and the labile iron pool (LIP) in sepsis-induced myocardial injury indicates that ferroptosis plays a role in sepsis. The regulatory mechanism of systemic inflammation and the immune response in MAPK-related ferroptosis needs to be further studied.

It has been reported that noncoding RNAs (including miRNAs and lncRNAs) play significant roles in the diagnosis, severity, and prognosis of septic patients (Wu et al., 2020). First, we identified 10 miRNAs that targeted more than one gene. Furthermore, 5 of these 10 miRNAs (hsa-miR-199a-3p, hsa-miR-17-5p, hsa-miR-106a-5p, hsa-let-7b-5p and hsa-miR-214-3p) predicted upstream lncRNAs. In previous studies, miR-199a-3p has been identified as a potential biomarker of neonatal sepsis (Abdelaleem et al., 2022). miR-17-5p and miR-106a-5p participate in sepsis-associated cardiomyopathy or acute kidney injury (AKI) by regulating oxidative stress *via* high mobility group box (HMBG) 1 (Qiu et al., 2021; Xu, Ma & Yang, 2021). miR-214-3p attenuates sepsis-induced myocardial dysfunction by inhibiting autophagy (Sang et al., 2020). Let-7b-5p was significantly downregulated in renal tubular epithelial cells during sepsis-induced AKI (Gao et al., 2020). In terms of upstream lncRNAs, taurine-upregulated gene (TUG) one was downregulated and NEAT1 was upregulated in septic patients (Gao et al., 2020; Wang et al., 2020). Our results suggest the roles of the mRNA–miRNA–lncRNA network in sepsis. Previous studies have demonstrated that the noncoding RNAs let-7b-5p and NEAT1 participate in ferroptosis by regulating the MAPK signaling pathway (Chen, Xin & Zhang, 2019; Du et al., 2019). Combined with our gene enrichment analysis results, the MAPK-related mRNA–miRNA–lncRNA network may regulate ferroptosis during sepsis. The latest study found that the ferroptosis-related gene LPIN1, which is associated with the immune status, could become a reliable biomarker of patient survival in sepsis (Dai et al., 2022). To verify the changes in the above screened ferroptosis-related key mRNAs, miRNAs and lncRNAs in the immune system, we next detected the expression of MAPK14, DUSP1, MAP3K5, MAPK1, MAPK8, let-7b-5p and NEAT1 in peripheral blood leukocytes from septic mice during the acute inflammatory phase. The upregulation of MAPK14, MAP3K5, MAPK1, MAPK8 and NETA1 indicated their important roles in promoting acute systemic inflammation. In contrast, the downregulation of DUSP1 and let-7b-5p demonstrated their protective roles during acute inflammation. Notably, the mRNA expression of DUSP1 and MAPK8 is contradictory between the human datasets and the CLP mouse model. One possible reason is that the types of samples detected are different. In the human datasets, the array-based gene expression profiles were obtained from whole blood, including plasma, blood cells, platelets, etc. In the CLP mouse model, we extracted the total RNA from peripheral blood leukocytes in mice, which included lymphocytes, neutrophils, monocytes, etc. Thus, it is possible that DUSP1 and MAPK8 play different biological roles in immune cells and whole blood, and further studies are needed. Despite the regulatory network of MAPK molecules involved in ferroptosis has been shown to be changed in sepsis, the specific mechanisms of MAPK- related ferroptosis in sepsis-induced immune dysregulation and systemic inflammation need to be further studied.

There are still several limitations in the present study. First, the data we analyzed were downloaded from GEO datasets, and further prospective clinical studies are necessary to validate the observations. Second, the potential mRNA–miRNA–lncRNA network we indicated needs further experimental validation. In addition, with an in-depth study of ferroptosis, basic experimental studies are needed to explore the relationship between ferroptosis and sepsis.

## Conclusion

The MAPK signaling pathway may play a key role in regulating ferroptosis during sepsis. This study provides a valuable resource for future studies investigating the mechanism of MAPK-related ferroptosis in sepsis. More comprehensive and in-depth studies should be conducted to explore the associations between ferroptosis-related genes and sepsis-induced immune dysregulation and systemic inflammation.

## Supplemental Information

10.7717/peerj.13983/supp-1 Supplemental Information 1 Overview of the GEO data. The clustering of sepsis patient and control group of samples are significantly separated. UMAP dimension reduction shows segregation the two groups in GSE13904 (A) and in GSE28750 (B). (C) PCA of the gene expression profiles in GSE13904. Two principal components containing 44.0% of the variance. (D) PCA plot of the gene expression profiles in GSE28750. Two principal components containing 59.1% of the variance. The boxplots show that the signal intensity of each sample is almost at the same median level in GSE13904 (E) and in GSE28750 (F).

10.7717/peerj.13983/supp-2 Supplemental Information 2 Histopathological analysis for multiorgans. Twenty-four hours after the CLP procedure, the lung, liver and renal tissues from the CLP mice and the sham group were collected. Representative images of lung, liver and kidney stained with hematoxylin and eosin. In CLP group, the injured lung tissues of mice exhibit alveolar congestion, hemorrhage and alveolar wall thickening. The liver shows inflammatory cell infiltration and necrosis. And the injured kidney exhibit disordered epithelium and interstitial edema.

10.7717/peerj.13983/supp-3 Supplemental Information 3 A list of human expressed ferroptosis-related genes.

10.7717/peerj.13983/supp-4 Supplemental Information 4 The sequences of primers.

10.7717/peerj.13983/supp-5 Supplemental Information 5 A list of 53 ferroptosis-related DEGs in the GSE13904 dataset.

10.7717/peerj.13983/supp-6 Supplemental Information 6 A list of 75 ferroptosis-related DEGs in the GSE28750 dataset.

10.7717/peerj.13983/supp-7 Supplemental Information 7 Summary of GO (BP, CC and MF analysis) and KEGG pathway terms with the number of enriched genes.

10.7717/peerj.13983/supp-8 Supplemental Information 8 A list of 27 genes and their interaction scores in PPI network.

10.7717/peerj.13983/supp-9 Supplemental Information 9 The full MCC scores of 27 interaction proteins.

10.7717/peerj.13983/supp-10 Supplemental Information 10 Summary of top 8 GO and KEGG pathways of the key clustering module.

10.7717/peerj.13983/supp-11 Supplemental Information 11 A total of 168 miRNAs targeting 6 genes.

10.7717/peerj.13983/supp-12 Supplemental Information 12 Summary of lncRNAs predicted scores and the correlation with targeting miRNAs.

10.7717/peerj.13983/supp-13 Supplemental Information 13 PCR.

10.7717/peerj.13983/supp-14 Supplemental Information 14 Randomisation sequence for mice grouping.

10.7717/peerj.13983/supp-15 Supplemental Information 15 Author checklist.

## Additional Information and Declarations

### Data Availability

The following information was supplied regarding data availability: The raw measurements are available in the Supplemental Files.
