# Supplementary figures and images for: Screening of potential key ferroptosis-related genes in sepsis
Source: PeerJ. 2022 Sep 13;10:e13983. doi: 10.7717/peerj.13983 (PMC9480065; doi:10.7717/peerj.13983)

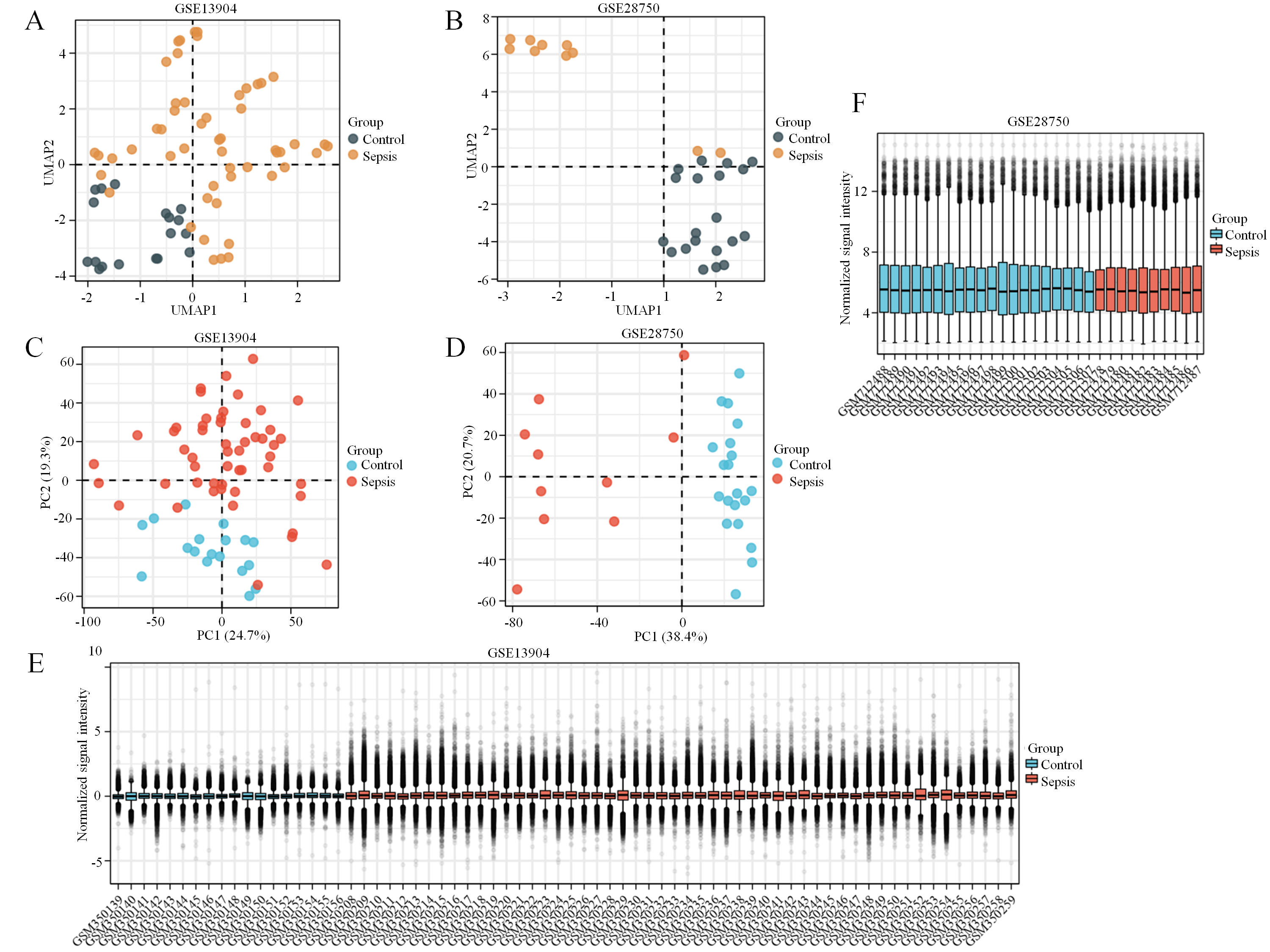

Supplement: Supplemental Information 1 — The clustering of sepsis patient and control group of samples are significantly separated. UMAP dimension reduction shows segregation the two groups in GSE13904 (A) and in GSE28750 (B). (C) PCA of the gene expression profiles in GSE13904. Two principal components containing 44.0% of the variance. (D) PCA plot of the gene expression profiles in GSE28750. Two principal components containing 59.1% of the variance. The boxplots show that the signal intensity of each sample is almost at the same median level in GSE13904 (E) and in GSE28750 (F). [file peerj-10-13983-s001.png]

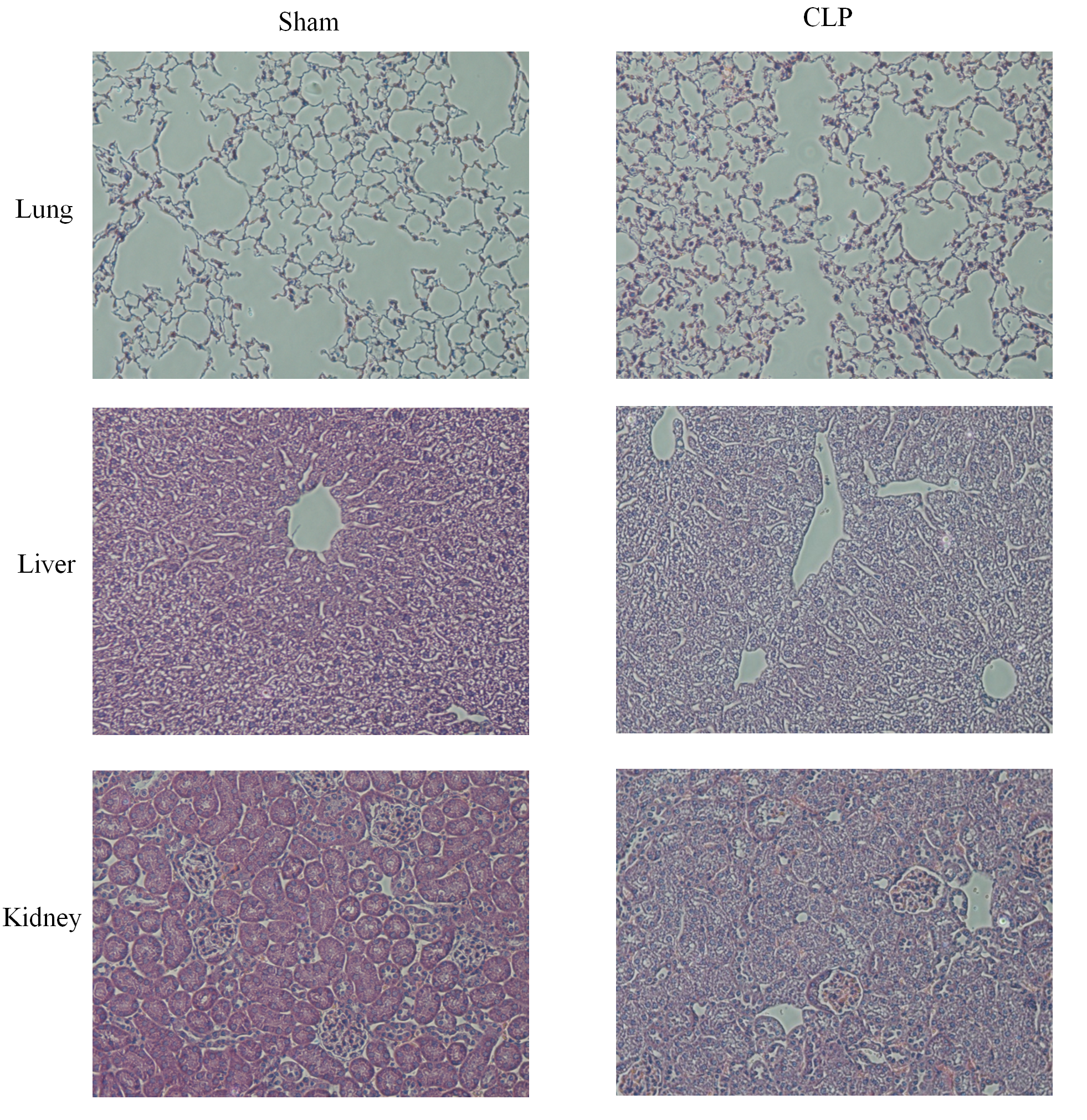

Supplement: Supplemental Information 2 — Twenty-four hours after the CLP procedure, the lung, liver and renal tissues from the CLP mice and the sham group were collected. Representative images of lung, liver and kidney stained with hematoxylin and eosin. In CLP group, the injured lung tissues of mice exhibit alveolar congestion, hemorrhage and alveolar wall thickening. The liver shows inflammatory cell infiltration and necrosis. And the injured kidney exhibit disordered epithelium and interstitial edema. [file peerj-10-13983-s002.png]
