# Supplementary material for: Screening of potential key ferroptosis-related genes in sepsis
Source: PeerJ. 2022 Sep 13;10:e13983. doi: 10.7717/peerj.13983 (PMC9480065; doi:10.7717/peerj.13983)
Supplement: Supplemental Information 3 [file peerj-10-13983-s003.pdf]

Gene symbol	Gene name	Role in ferroptosis
ACO1	Aconitase 1	driver
ACSF2	Acyl-CoA synthetase family member 2	driver
ACSL4	Acyl-CoA synthetase long chain family member 4	driver
ACVR1B	Activin A receptor type 1B	driver
ALOX12	Arachidonate 12-lipoxygenase, 12S type	driver
ALOX12B	Arachidonate 12-lipoxygenase, 12R type	driver
ALOX15	Arachidonate 15-lipoxygenase	driver
ALOX15B	Arachidonate 15-lipoxygenase type B	driver
ALOX5	Arachidonate 5-lipoxygenase	driver
ALOXE3	Arachidonate lipoxygenase 3	driver
ANO6	Anoctamin 6	driver
ATF3	Activating transcription factor 3	driver
ATG13	Autophagy related 13	driver
ATG16L1	Autophagy related 16 like 1	driver
ATG3	Autophagy related 3	driver
ATG4D	Autophagy related 4D cysteine peptidase	driver
ATG5	Autophagy related 5	driver
ATG7	Autophagy related 7	driver

ATM	ATM serine/threonine kinase	driver
ATP5MC3	ATP synthase membrane subunit c locus 3	driver
BACH1	BTB domain and CNC homolog 1	driver
BAP1	BRCA1 associated protein 1	driver
BECN1	Beclin 1	driver
BID	BH3 interacting domain death agonist	driver
CARS1	Cysteinyl-tRNA synthetase 1	driver
CDKN2A	Cyclin dependent kinase inhibitor 2A	driver
CDO1	Cysteine dioxygenase type 1	driver
CHAC1	ChaC glutathione specific gamma-glutamylcyclotransferase 1	driver
CS	Citrate synthase	driver
CYBB	Cytochrome b-245 beta chain	driver
DNAJB6	DnaJ heat shock protein family (Hsp40) member B6	driver
DPP4	Dipeptidyl peptidase 4	driver
DUOX1	Dual oxidase 1	driver
DUOX2	Dual oxidase 2	driver
EGFR	Epidermal growth factor receptor	driver
EGLN2	Egl-9 family hypoxia inducible factor 2	driver

ELAVL1	ELAV like RNA binding protein 1	driver
EMC2	ER membrane protein complex subunit 2	driver
EPAS1	Endothelial PAS domain protein 1	driver
FBXW7	F-box and WD repeat domain containing 7	driver
FLT3	Fms related tyrosine kinase 3	driver
G6PD	Glucose-6-phosphate dehydrogenase	driver
G6PDX	_NA_	driver
GABARAPL1	GABA type A receptor associated protein like 1	driver
GABARAPL2	GABA type A receptor associated protein like 2	driver
GLS2	Glutaminase 2	driver
GOT1	Glutamic-oxaloacetic transaminase 1	driver
HIF1A	Hypoxia inducible factor 1 subunit alpha	driver
HILPDA	Hypoxia inducible lipid droplet associated	driver
HMGB1	High mobility group box 1	driver
HMOX1	Heme oxygenase 1	driver
HRAS	HRas proto-oncogene, GTPase	driver
IDH1	Isocitrate dehydrogenase (NADP(+)) 1	driver
IFNG	Interferon gamma	driver
IREB2	Iron response element binding protein 2	driver
KEAP1	Kelch like ECH associated protein 1	driver

KRAS	KRAS proto-oncogene, GTPase	driver
LINC00472	Long intergenic non-protein coding RNA 472	driver
LONP1	Lon peptidase 1, mitochondrial	driver
LPCAT3	Lysophosphatidylcholine acyltransferase 3	driver
LPIN1	Lipin 1	driver
MAP1LC3A	Microtubule associated protein 1 light chain 3 alpha	driver
MAPK1	Mitogen-activated protein kinase 1	driver
MAPK14	Mitogen-activated protein kinase 14	driver
MAPK3	Mitogen-activated protein kinase 3	driver
MAPK8	Mitogen-activated protein kinase 8	driver
MAPK9	Mitogen-activated protein kinase 9	driver
MIOX	Myo-inositol oxygenase	driver
MIR6852	microRNA 6852	driver
MTDH	Metadherin	driver
MYB	MYB proto-oncogene, transcription factor	driver
NCOA4	Nuclear receptor coactivator 4	driver
NOX1	Nicotinamide adenine dinucleotide phosphate (NADPH) oxidase (NOX) 1	driver
NOX3	Nicotinamide adenine dinucleotide phosphate	driver

	(NADPH) oxidase (NOX) 3	
NOX4	Nicotinamide adenine dinucleotide phosphate (NADPH) oxidase (NOX) 4	driver
NOX5	Nicotinamide adenine dinucleotide phosphate (NADPH) oxidase (NOX) 5	driver
NRAS	NRAS proto-oncogene, GTPase	driver
PANX1	Pannexin 1	driver
PEBP1	Phosphatidylethanolamine binding protein 1	driver
PGD	Phosphoglycerate dehydrogenase	driver
PHKG2	Phosphorylase kinase catalytic subunit gamma 2	driver
PIK3CA	Phosphatidylinositol-4,5-bisphosphate 3-kinase catalytic subunit alpha	driver
PRKAA1	Protein kinase AMP-activated catalytic subunit alpha 1	driver
PRKAA2	Protein kinase AMP-activated catalytic subunit alpha 2	driver
RPL8	Ribosomal protein L8	driver
SAT1	Spermidine/spermine N1-acetyltransferase 1	driver
SCP2	Sterol carrier protein 2	driver
SIRT1	Sirtuin 1	driver

SLC1A5	Solute carrier family 1 member 5	driver
SLC38A1	Solute carrier family 38 member 1	driver
SNX4	Sorting nexin 4	driver
SOCS1	Suppressor of cytokine signaling 1	driver
TAZ	Tafazzin	driver
TF	Transferrin	driver
TFR2	Transferrin receptor 2	driver
TFRC	Transferrin receptor	driver
TGFBR1	Transforming growth factor beta receptor 1	driver
TLR4	Toll like receptor 4	driver
TNFAIP3	TNF alpha induced protein 3	driver
TP53	Tumor protein p53	driver
ULK1	Unc-51 like autophagy activating kinase 1	driver
ULK2	Unc-51 like autophagy activating kinase 2	driver
VDAC2	Valtage-dependent anion channels 2	driver
WIPI1	WD repeat domain, phosphoinositide interacting 1	driver
WIPI2	WD repeat domain, phosphoinositide interacting 2	driver
YY1AP1	YY1 associated protein 1	driver
ZEB1	Zinc finger E-box binding homeobox 1	driver
AGPAT3	1-acylglycerol-3-phosphate O-acyltransferase 3	marker

ALB	Albumin	marker
ANGPTL7	Angiopoietin like 7	marker
ARRDC3	Arrestin domain containing 3	marker
ASNS	Asparagine synthetase (glutamine-hydrolyzing)	marker
ATF4	Activating transcription factor 4	marker
ATP6V1G2	ATPase H+ transporting V1 subunit G2	marker
AURKA	Aurora kinase A	marker
BLOC1S5-TXNDC5	BLOC1S5-TXNDC5 readthrough (NMD candidate)	marker
BNIP3	BCL2 interacting protein 3	marker
CAPG	Capping actin protein, gelsolin like	marker
CBS	Cystathionine beta-synthase	marker
CEBPG	CCAAT enhancer binding protein gamma	marker
CXCL2	C-X-C motif chemokine ligand 2	marker
DDIT3	DNA damage inducible transcript 3	marker
DDIT4	DNA damage inducible transcript 4	marker
DRD4	Dopamine receptor D4	marker
DRD5	Dopamine receptor D5	marker
DUSP1	Dual specificity phosphatase 1	marker
EIF2AK4	Eukaryotic translation initiation factor 2 alpha	marker

	kinase 4	
EIF2S1	Eukaryotic translation initiation factor 2 subunit 1	marker
FTH1	Ferritin heavy chain 1	marker
FTL	Ferritin light chain	marker
GABPB1	GA binding protein transcription factor subunit beta 1	marker
GDF15	Growth differentiation factor 15	marker
GPT2	Glutamic--pyruvic transaminase 2	marker
GPX2	Glutathione peroxidase 2	marker
GPX4	Glutathione peroxidase 4	marker
HAMP	Hepcidin antimicrobial peptide	marker
HBA1	Hemoglobin subunit alpha 1	marker
HERPUD1	Homocysteine inducible ER protein with ubiquitin like domain 1	marker
HIC1	HIC ZBTB transcriptional repressor 1	marker
HNF4A	Hepatocyte nuclear factor 4 alpha	marker
HSD17B11	Hydroxysteroid 17-beta dehydrogenase 11	marker
HSPB1	Heat shock protein family B (small) member 1	marker
IL33	Interleukin 33	marker
IL6	Interleukin 6	marker

JDP2	Jun dimerization protein 2	marker
KLHL24	Kelch like family member 24	marker
LURAP1L	Leucine rich adaptor protein 1 like	marker
MAFG	MAF bZIP transcription factor G	marker
MAP3K5	Mitogen-activated protein kinase kinase kinase 5	marker
MIR30B	microRNA 30b	marker
MIR4715	microRNA 4715	marker
MT3	Metallothionein 3	marker
NCF2	Neutrophil cytosolic factor 2	marker
NFE2L2	Nuclear factor, erythroid 2 like 2	marker
NGB	Neuroglobin	marker
NNMT	Nicotinamide N-methyltransferase	marker
NOS2	Nitric oxide synthase 2	marker
OXSR1	Oxidative stress responsive kinase 1	marker
PCK2	Phosphoenolpyruvate carboxykinase 2, mitochondrial	marker
PLIN4	Perilipin 4	marker
PRDX1	Peroxiredoxin 1	marker
PSAT1	Phosphoserine aminotransferase 1	marker
PTGS2	Prostaglandin-endoperoxide synthase 2	marker

RELA	RELA proto-oncogene, NF-kB subunit	marker
RGS4	Regulator of G protein signaling 4	marker
RIPK1	Receptor interacting serine/threonine kinase 1	marker
RRM2	Ribonucleotide reductase regulatory subunit M2	marker
SELENOS	Selenoprotein S	marker
SESN2	Sestrin 2	marker
SETD1B	SET domain containing 1B, histone lysine methyltransferase	marker
SLC1A4	Solute carrier family 1 member 4	marker
SLC2A1	Solute carrier family 2 member 1	marker
SLC2A12	Solute carrier family 2 member 12	marker
SLC2A14	Solute carrier family 2 member 14	marker
SLC2A3	Solute carrier family 2 member 3	marker
SLC2A6	Solute carrier family 2 member 6	marker
SLC2A8	Solute carrier family 2 member 8	marker
SLC3A2	Solute carrier family 3 member 2	marker
SLC40A1	Solute carrier family 40 member 1	marker
SLC7A11	Solute carrier family 7 member 11	marker
SLC7A5	Solute carrier family 7 member 5	marker
SNORA16A	Small nucleolar RNA, H/ACA box 16A	marker

SP1	Sp1 transcription factor	marker
SRXN1	Sulfiredoxin 1	marker
STEAP3	STEAP3 metalloreductase	marker
STMN1	Stathmin 1	marker
TFAP2C	Transcription factor AP-2 gamma	marker
TRIB3	Tribbles pseudokinase 3	marker
TSC22D3	TSC22 domain family member 3	marker
TUBE1	Tubulin epsilon 1	marker
TXNIP	Thioredoxin interacting protein	marker
TXNRD1	Thioredoxin reductase 1	marker
UBC	Ubiquitin C	marker
VEGFA	Vascular endothelial growth factor A	marker
VLDLR	Very low density lipoprotein receptor	marker
XBP1	X-box binding protein 1	marker
YWHAE	Tyrosine 3-monooxygenase/tryptophan 5-monooxygenase activation protein epsilon	marker
ZFP69B	ZFP69 zinc finger protein B	marker
ZNF419	Zinc finger protein 419	marker
ACSL3	Acyl-CoA synthetase long chain family member 3	suppressor
AIFM2	Apoptosis inducing factor mitochondria associated	suppressor

	2	
AKR1C1	Aldo-keto reductase family 1 member C1	suppressor
AKR1C2	Aldo-keto reductase family 1 member C2	suppressor
AKR1C3	Aldo-keto reductase family 1 member C3	suppressor
ARNTL	Aryl hydrocarbon receptor nuclear translocator like	suppressor
BRD4	Bromodomain containing 4	suppressor
CA9	Carbonic anhydrase 9	suppressor
CAV1	Caveolin 1	suppressor
CD44	CD44 molecule (Indian blood group)	suppressor
CDKN1A	Cyclin dependent kinase inhibitor 1A	suppressor
CHMP5	Charged multivesicular body protein 5	suppressor
CHMP6	Charged multivesicular body protein 6	suppressor
CISD1	CDGSH iron sulfur domain 1	suppressor
CISD2	CDGSH iron sulfur domain 2	suppressor
FADS2	Fatty acid desaturase 2	suppressor
FH	Fumarate hydratase	suppressor
GCH1	GTP cyclohydrolase 1	suppressor
HELLS	Helicase, lymphoid specific	suppressor
HSF1	Heat shock transcription factor 1	suppressor

HSPA5	Heat shock protein family A (Hsp70) member 5	suppressor
ISCU	Iron-sulfur cluster assembly enzyme	suppressor
JUN	Jun proto-oncogene, AP-1 transcription factor subunit	suppressor
LAMP2	Lysosomal associated membrane protein 2	suppressor
LINC00336	Long intergenic non-protein coding RNA 336	suppressor
MIR137	microRNA 137	suppressor
MIR17	microRNA 17	suppressor
MIR9-1	microRNA 9-1	suppressor
MIR9-2	microRNA 9-2	suppressor
MIR9-3	microRNA 9-3	suppressor
MT1G	Metallothionein 1G	suppressor
MUC1	Mucin 1, cell surface associated	suppressor
NF2	Neurofibromin 2	suppressor
NFS1	NFS1 cysteine desulfurase	suppressor
NQO1	NAD(P)H quinone dehydrogenase 1	suppressor
OTUB1	OTU deubiquitinase, ubiquitin aldehyde binding 1	suppressor
PLIN2	Perilipin 2	suppressor
PML	Promyelocytic leukemia	suppressor
PRDX6	Peroxiredoxin 6	suppressor

PROM2	Prominin 2	suppressor
RB1	RB transcriptional corepressor 1	suppressor
SCD	Stearoyl-CoA desaturase	suppressor
SQSTM1	Sequestosome 1	suppressor
SRC	SRC proto-oncogene, non-receptor tyrosine kinase	suppressor
STAT3	Signal transducer and activator of transcription 3	suppressor
TMBIM4	Transmembrane BAX inhibitor motif containing 4	suppressor
TP63	Tumor protein p63	suppressor
ZFP36	ZFP36 ring finger protein	suppressor
