# Supplementary material for: Screening of potential key ferroptosis-related genes in sepsis
Source: PeerJ. 2022 Sep 13;10:e13983. doi: 10.7717/peerj.13983 (PMC9480065; doi:10.7717/peerj.13983)
Supplement: Supplemental Information 4 [file peerj-10-13983-s004.pdf]

name	forward (5'-3')	reverse (5'-3')
MAPK14	CGCTTTGTTGACTTCCAACC	TTTGACGATGTTGTCGTGGT
DUSP1	GTTGTTGGATTGTCGCTCCTT	TTGGGCACGATATGCTCCAG
MAP3K5	TTTGTTTCGTGAGACTGCGTACC	AGACACTTGGGCACACTACACA
MAPK1	TGCTTTCTCTCCCGCACAAA	TGAATGGCGCTTCAGCAATG
MAPK8	TTCCCAGCTGACTCAGAGCATAAC	TAGTCATCTACAGCAGCCCAGAGG
NEAT1	CTTCCTCCCTTTAACTTATCCATTCAC	CTCTTCCTCCACCATTACCAACAATAC
let-7b-5p	GGGTGAGGTAGTAGGTTGT	GTTGGCTCTGGTGCAGGGTCCGAGGTATTCGCACCAGAGCCAACAACCAC
	the universal reverse PCR primer, 5′-GTGCAGGGTCCGAGGT-3′	
