# Supplementary material for: Screening of potential key ferroptosis-related genes in sepsis
Source: PeerJ. 2022 Sep 13;10:e13983. doi: 10.7717/peerj.13983 (PMC9480065; doi:10.7717/peerj.13983)
Supplement: Supplemental Information 5 [file peerj-10-13983-s005.pdf]

	Gene symbol	adj.p.value	logFC	Gene title	ID
UP regulated genes	SLC2A3	3.71E-09	2.090	solute carrier family 2 member 3	202498_s_at
	PGD	1.48E-09	2.060	phosphogluconate dehydrogenase	201118_at
	MAPK14	9.67E-09	2.010	mitogen-activated protein kinase 14	210449_x_at
	ALOX5	0.00000207	1.470	arachidonate 5-lipoxygenase	214366_s_at
	WIPI1	0.00000643	1.270	WD repeat domain, phosphoinositide interacting 1	213836_s_at
	G6PD	0.0000539	1.130	glucose-6-phosphate dehydrogenase	202275_at
	JDP2	0.0000205	1.130	Jun dimerization protein 2	226267_at
	SLC40A1	0.00000178	1.110	solute carrier family 40 member 1	223044_at
	TLR4	0.00732	1.110	toll like receptor 4	232068_s_at
	ACSL4	0.00126	1.100	acyl-CoA synthetase long-chain family member 4	202422_s_at
	LAMP2	0.000177	1.070	lysosomal associated membrane	203041_s_at

			protein 2	
ATG7	0.000000484	1.040	autophagy related 7	218673_s_at
MAPK1	0.000000531	1.030	mitogen-activated protein kinase 1	212271_at
SP1	0.0039	1.010	Sp1 transcription factor	1553685_s_at
MAFG	3.95E-09	0.991	MAF bZIP transcription factor G	204970_s_at
CD44	0.00346	0.98	CD44 molecule (Indian blood group)	1565868_at
STAT3	0.00000384	0.953	signal transducer and activator of transcription 3	208992_s_at
ACVR1B	0.00000112	0.947	activin A receptor type 1B	213198_at
YWHAE	0.00114	0.932	tyrosine 3-monooxygenase/tryptophan 5-monooxygenase activation protein epsilon	210317_s_at
IDH1	0.000306	0.905	isocitrate dehydrogenase (NADP(+)) 1, cytosolic	1555037_a_at
CAPG	0.000146	0.890	capping actin protein, gelsolin like	201850_at
MAP3K5	0.0000586	0.878	mitogen-activated protein kinase	203836_s_at

			kinase kinase 5	
SAT1	0.00000336	0.864	spermidine/spermine N1-acetyltransferase 1	213988_s_at
FLT3	0.000299	0.834	fms related tyrosine kinase 3	206674_at
NCF2	0.0000686	0.833	neutrophil cytosolic factor 2	209949_at
CYBB	0.00167	0.826	cytochrome b-245 beta chain	203922_s_at
BID	0.00275	0.811	BH3 interacting domain death agonist	204493_at
EPAS1	0.00174	0.758	endothelial PAS domain protein 1	200878_at
ZFP36	0.000701	0.653	ZFP36 ring finger protein	201531_at
GABARAPL2	0.000531	0.620	GABA type A receptor associated protein like 2	209046_s_at
SLC7A5	0.0278	0.604	solute carrier family 7 member 5	201195_s_at
DUSP1	0.0202	0.550	dual specificity phosphatase 1	201044_x_at
HMOX1	0.0291	0.536	heme oxygenase 1	203665_at
BACH1	0.0388	0.534	BTB domain and CNC homolog 1	204194_at
FTH1	0.0000905	0.508	ferritin heavy chain 1	200748_s_at
TXNRD1	0.00987	0.506	thioredoxin reductase 1	201266_at

	CBS	0.00699	0.503	cystathionine-beta-synthase	1553972_a_at
	SRXN1	0.0223	0.502	sulfiredoxin 1	225252_at
Down regulated gene	SLC38A1	0.0000437	-1.390	solute carrier family 38 member 1	224579_at
	ATM	0.00005	-1.190	ATM serine/threonine kinase	212672_at
	AKR1C3	0.000277	-1.030	aldo-keto reductase family 1, member C3	209160_at
	TUBE1	0.00821	-0.924	tubulin epsilon 1	226181_at
	LPIN1	0.000328	-0.919	lipin 1	212276_at
	PEBP1	0.000409	-0.901	phosphatidylethanolamine binding protein 1	210825_s_at
	MAPK8	0.0000263	-0.770	mitogen-activated protein kinase 8	229664_at
	ZEB1	0.000535	-0.736	zinc finger E-box binding homeobox 1	212764_at
	DPP4	0.021	-0.676	dipeptidyl peptidase 4	211478_s_at
	SNX4	0.00708	-0.654	sorting nexin 4	212652_s_at
	SIRT1	0.00287	-0.647	sirtuin 1	218878_s_at
	ARRDC3	0.0078	-0.642	arrestin domain containing 3	224797_at

	BNIP3	0.0418	-0.631	BCL2 interacting protein 3	201849_at
	SCP2	0.0204	-0.595	sterol carrier protein 2	211733_x_at
	KLHL24	0.0424	-0.573	kelch like family member 24	226158_at
