# Supplementary material for: Screening of potential key ferroptosis-related genes in sepsis
Source: PeerJ. 2022 Sep 13;10:e13983. doi: 10.7717/peerj.13983 (PMC9480065; doi:10.7717/peerj.13983)
Supplement: Supplemental Information 6 [file peerj-10-13983-s006.pdf]

	Gene symbol	adj.p.value	logFC	Gene title	ID
Up regulated genes	ACSL4	6.62E-06	2.289	acyl-CoA synthetase long-chain family member 4	202422_s_at
	RRM2	2.15E-05	2.135	ribonucleotide reductase regulatory subunit M2	201890_at
	SLC2A3	1.97E-08	1.987	solute carrier family 2 member 3	202499_s_at
	MAPK14	2.5E-06	1.758	mitogen-activated protein kinase 14	211561_x_at
	CISD2	6.85E-07	1.717	CDGSH iron sulfur domain 2	226686_at
	WIPI1	4.66E-06	1.706	WD repeat domain, phosphoinositide interacting 1	213836_s_at
	SLC40A1	5.18E-06	1.633	solute carrier family 40 member 1	223044_at
	PGD	8.13E-06	1.597	phosphogluconate dehydrogenase	201118_at
	EMC2	0.000497	1.544	ER membrane protein complex subunit 2	203584_at
	ACSL3	1.37E-06	1.486	acyl-CoA synthetase long-chain family member 3	201660_at
	CHMP5	0.000524	1.472	charged multivesicular body protein 5	218085_at
	EPAS1	0.000095	1.461	endothelial PAS domain protein 1	200878_at

CAPG	3.38E-08	1.379	capping actin protein, gelsolin like	201850_at
CYBB	0.000034	1.285	cytochrome b-245 beta chain	203923_s_at
IDH1	3.83E-07	1.282	isocitrate dehydrogenase (NADP(+)) 1, cytosolic	201193_at
TGFBR1	0.00149	1.269	transforming growth factor beta receptor 1	224793_s_at
TFRC	0.00123	1.247	transferrin receptor	208691_at
RB1	0.000558	1.180	RB transcriptional corepressor 1	203132_at
ACVR1B	4.66E-06	1.160	activin A receptor type 1B	213198_at
NRAS	0.000182	1.143	neuroblastoma RAS viral oncogene homolog	202647_s_at
MAFG	7.89E-09	1.129	MAF bZIP transcription factor G	204970_s_at
PRKAA1	0.00245	1.059	protein kinase AMP-activated catalytic subunit alpha 1	225984_at
CD44	0.000645	1.000	CD44 molecule (Indian blood group)	217523_at
ALOX5	2.75E-06	0.999	arachidonate 5-lipoxygenase	204446_s_at
AURKA	0.000985	0.972	aurora kinase A	208079_s_at
MTDH	0.0125	0.971	metadherin	212250_at
TLR4	0.0297	0.957	toll like receptor 4	232068_s_at
HIF1A	0.0072	0.913	hypoxia inducible factor 1 alpha	200989_at

			subunit	
GABARAPL2	2.28E-05	0.900	GABA type A receptor associated protein like 2	209046_s_at
PSAT1	0.0151	0.851	phosphoserine aminotransferase 1	223062_s_at
GCH1	0.00578	0.851	GTP cyclohydrolase 1	204224_s_at
MAPK1	3.13E-06	0.841	mitogen-activated protein kinase 1	212271_at
JDP2	6.58E-05	0.840	Jun dimerization protein 2	226267_at
SAT1	0.00012	0.838	spermidine/spermine N1-acetyltransferase 1	203455_s_at
SLC7A5	0.00392	0.777	solute carrier family 7 member 5	201195_s_at
LAMP2	0.000994	0.708	lysosomal associated membrane protein 2	200821_at
VEGFA	0.0315	0.685	vascular endothelial growth factor A	210512_s_at
CBS	0.00193	0.674	cystathionine-beta-synthase	1553972_a_at
DUSP1	0.0444	0.667	dual specificity phosphatase 1	201041_s_at
KRAS	0.000804	0.667	KRAS proto-oncogene, GTPase	204009_s_at
ATG3	0.000106	0.663	autophagy related 3	220237_at
SESN2	0.0179	0.637	sestrin 2	223196_s_at
SNX4	0.0186	0.598	sorting nexin 4	205329_s_at
DNAJB6	0.00352	0.594	DnaJ heat shock protein family	209015_s_at

				(Hsp40) member B6	
	SLC2A1	0.00178	0.577	solute carrier family 2 member 1	201250_s_at
	G6PD	0.0445	0.560	glucose-6-phosphate dehydrogenase	202275_at
	FLT3	0.000312	0.559	fms related tyrosine kinase 3	206674_at
	SLC7A11	3.34E-05	0.533	solute carrier family 7 member 11	209921_at
	MAP3K5	0.00906	0.529	mitogen-activated protein kinase kinase kinase 5	203837_at
	GDF15	0.00684	0.523	growth differentiation factor 15	221577_x_at
Down regulated genes	SLC38A1	1.15E-06	-2.008	solute carrier family 38 member 1	218237_s_at
	PEBP1	2.41E-07	-1.724	phosphatidylethanolamine binding protein 1	210825_s_at
	TP53	1.89E-07	-1.350	tumor protein p53	201746_at
	LPIN1	1.35E-05	-1.266	lipin 1	212274_at
	TXNIP	0.0082	-1.118	thioredoxin interacting protein	201008_s_at
	DPP4	1.05E-05	-1.114	dipeptidyl peptidase 4	211478_s_at
	ATM	0.00241	-1.093	ATM serine/threonine kinase	210858_x_at
	RPL8	1.97E-05	-0.953	ribosomal protein L8	200936_at
	AKR1C3	0.0139	-0.938	aldo-keto reductase family 1,	209160_at

			member C3	
ELAVL1	0.000164	-0.775	ELAV like RNA binding protein 1	227746_at
ATG16L1	0.000556	-0.762	autophagy related 16 like 1	220521_s_at
LONP1	2.27E-05	-0.761	lon peptidase 1, mitochondrial	209017_s_at
ZNF419	1.81E-05	-0.733	zinc finger protein 419	219826_at
CS	2.83E-05	-0.652	citrate synthase	208660_at
MAPK8	0.000205	-0.623	mitogen-activated protein kinase 8	229664_at
BRD4	0.0249	-0.563	bromodomain containing 4	202102_s_at
AGPAT3	0.025	-0.552	1-acylglycerol-3-phosphate O-acyltransferase 3	223182_s_at
ZEB1	0.0133	-0.542	zinc finger E-box binding homeobox 1	212758_s_at
RELA	2.86E-05	-0.538	RELA proto-oncogene, NF-kB subunit	201783_s_at
HERPUD1	0.00105	-0.536	homocysteine inducible ER protein with ubiquitin like domain 1	217168_s_at
SQSTM1	0.0007	-0.531	sequestosome 1	201471_s_at
ACSF2	3.48E-05	-0.518	acyl-CoA synthetase family member 2	218844_at
EIF2AK4	0.0231	-0.511	eukaryotic translation initiation	225164_s_at

				factor 2 alpha kinase 4	
	WIPI2	0.00128	-0.508	WD repeat domain, phosphoinositide interacting 2	204710_s_at
	FH	0.00155	-0.500	fumarate hydratase	214170_x_at
