# Supplementary material for: Screening of potential key ferroptosis-related genes in sepsis
Source: PeerJ. 2022 Sep 13;10:e13983. doi: 10.7717/peerj.13983 (PMC9480065; doi:10.7717/peerj.13983)
Supplement: Supplemental Information 7 [file peerj-10-13983-s007.pdf]

Category	Term	Count	%	P.value
GOTERM_BP	positive regulation of transcription from RNA polymerase II promoter	8	23.52941176	0.002546543
GOTERM_BP	oxidation-reduction process	6	17.64705882	0.005610003
GOTERM_BP	peptidyl-serine phosphorylation	4	11.76470588	0.001866441
GOTERM_BP	positive regulation of apoptotic process	4	11.76470588	0.020742413
GOTERM_BP	activation of MAPK activity	4	11.76470588	0.018676979
GOTERM_BP	peptidyl-serine phosphorylation	4	11.76470588	0.001866441
GOTERM_BP	regulation of transcription from RNA polymerase II promoter	4	11.76470588	0.010513576
GOTERM_BP	protein phosphorylation	4	11.76470588	0.011778766
GOTERM_BP	positive regulation of gene expression	4	11.76470588	0.01450783
GOTERM_BP	activation of MAPK activity	4	11.76470588	0.018676979
GOTERM_BP	regulation of sequence-specific DNA binding transcription factor activity	3	8.823529412	0.001092241
GOTERM_BP	lipopolysaccharide-mediated	3	8.823529412	0.00179038

	signaling pathway			
GOTERM_BP	peptidyl-threonine phosphorylation	3	8.823529412	0.002518959
GOTERM_BP	response to nutrient	3	8.823529412	0.009260802
GOTERM_BP	cellular response to lipopolysaccharide	3	8.823529412	0.02068917
GOTERM_BP	protein autophosphorylation	3	8.823529412	0.044762732
GOTERM_BP	response to hypoxia	3	8.823529412	0.044762732
GOTERM_CC	cytosol	18	52.94117647	1.49E-05
GOTERM_CC	nucleus	17	50	0.017697554
GOTERM_CC	extracellular exosome	16	47.05882353	4.15E-05
GOTERM_CC	integral component of plasma membrane	9	26.47058824	0.003107806
GOTERM_CC	intracellular	7	20.58823529	0.001578994
GOTERM_CC	cytoplasm	7	20.58823529	0.030391157
GOTERM_CC	nuclear envelope	3	8.823529412	0.033471497
GOTERM_CC	autophagosome membrane	2	5.882352941	0.044328363
GOTERM_MF	ATP binding	9	26.47058824	0.006915887
GOTERM_MF	protein serine/threonine kinase activity	6	17.64705882	7.58E-04

GOTERM_MF	protein homodimerization activity	6	17.64705882	0.012958586
GOTERM_MF	enzyme binding	5	14.70588235	0.003874601
GOTERM_MF	MAP kinase activity	4	11.76471	3.32E-04
GOTERM_MF	protein binding	4	11.76471	0.004546126
GOTERM_MF	neutral amino acid transmembrane transporter activity	2	5.882352941	0.02130076
GOTERM_MF	glucose binding	2	5.882352941	0.02130076
GOTERM_MF	L-amino acid transmembrane transporter activity	2	5.882352941	0.025126064
KEGG_PATHWAY	Biosynthesis of antibiotics	4	11.76470588	0.049025507
KEGG_PATHWAY	Influenza A	4	11.76470588	0.029808597
KEGG_PATHWAY	MAPK signaling pathway	5	14.70588235	0.016101869
KEGG_PATHWAY	TNF signaling pathway	4	11.76470588	0.008158715
KEGG_PATHWAY	Toll-like receptor signaling pathway	4	11.76470588	0.007950633
KEGG_PATHWAY	Tuberculosis	5	14.70588235	0.004679737
KEGG_PATHWAY	Central carbon metabolism in cancer	4	11.76470588	0.001915754
KEGG_PATHWAY	Shigellosis	4	11.76470588	0.001915754

KEGG_PATHWAY	FoxO signaling pathway	5	14.70588235	0.001706101
KEGG_PATHWAY	Toxoplasmosis	5	14.70588235	8.18E-04
KEGG_PATHWAY	Proteoglycans in cancer	4	11.76470588	0.042420813
KEGG_PATHWAY	Osteoclast differentiation	4	11.76470588	0.014139835
KEGG_PATHWAY	Neurotrophin signaling pathway	4	11.76470588	0.011160041
KEGG_PATHWAY	Sphingolipid signaling pathway	4	11.76470588	0.011160041
KEGG_PATHWAY	Chagas disease (American trypanosomiasis)	4	11.76470588	0.007544059
KEGG_PATHWAY	Salmonella infection	4	11.76470588	0.00402044
KEGG_PATHWAY	Pertussis	4	11.76470588	0.003016994
