# Supplementary material for: Screening of potential key ferroptosis-related genes in sepsis
Source: PeerJ. 2022 Sep 13;10:e13983. doi: 10.7717/peerj.13983 (PMC9480065; doi:10.7717/peerj.13983)
Supplement: Supplemental Information 8 [file peerj-10-13983-s008.pdf]

Node1	Node2	Combined_score
MAPK14	DUSP1	0.998
PGD	G6PD	0.998
MAPK1	DUSP1	0.996
TLR4	CD44	0.989
MAPK1	MAPK14	0.977
DUSP1	MAPK8	0.974
MAPK1	PEBP1	0.967
MAPK1	MAPK8	0.931
GABARAPL2	WIPI1	0.898
MAP3K5	MAPK8	0.867
MAPK14	MAP3K5	0.863
MAPK14	MAPK8	0.803
FLT3	IDH1	0.802
MAPK14	CYBB	0.8
G6PD	IDH1	0.779
TLR4	CYBB	0.778
ATM	MAPK8	0.776
PGD	IDH1	0.77
SLC7A5	SLC38A1	0.768

ZEB1	CD44	0.713
TLR4	MAPK8	0.703
MAPK1	CYBB	0.689
MAPK14	TLR4	0.664
MAPK14	ATM	0.621
MAP3K5	TLR4	0.618
GABARAPL2	LAMP2	0.617
DPP4	CD44	0.599
JDP2	MAFG	0.533
SLC2A3	SLC38A1	0.532
DUSP1	TLR4	0.525
MAP3K5	CYBB	0.524
FLT3	CD44	0.519
ATM	IDH1	0.519
SLC2A3	G6PD	0.494
ALOX5	CYBB	0.493
TLR4	ALOX5	0.491
ATM	ZEB1	0.486
FLT3	ATM	0.475
JDP2	MAPK8	0.469

SLC2A3	SLC7A5	0.452
ACSL4	LPIN1	0.447
DUSP1	MAP3K5	0.437
WIPI1	LAMP2	0.43
MAPK1	ATM	0.429
MAPK14	JDP2	0.427
FLT3	TLR4	0.419
CYBB	MAPK8	0.404
