# Supplementary material for: Screening of potential key ferroptosis-related genes in sepsis
Source: PeerJ. 2022 Sep 13;10:e13983. doi: 10.7717/peerj.13983 (PMC9480065; doi:10.7717/peerj.13983)
Supplement: Supplemental Information 9 [file peerj-10-13983-s009.pdf]

RANK	MCC	Score
**1**	**MAPK14**	**68**
**2**	**MAPK8**	**68**
**3**	**TLR4**	**52**
**4**	**MAP3K5**	**48**
**5**	**CYBB**	**32**
**6**	**DUSP1**	**30**
**7**	**MAPK1**	**19**
**8**	**ATM**	**9**
9	FLT3	4
10	CD44	4
11	IDH1	4
12	JDP2	3
13	G6PD	3
14	SLC2A3	3
15	ALOX5	2
16	ZEB1	2
17	PGD	2
18	SLC38A1	2
19	SLC7A5	2

20	GABARAPL2	2
21	WIPI1	2
22	LAMP2	2
23	PEBP1	1
24	DPP4	1
25	MAFG	1
26	LPIN1	1
27	ACSL4	1
