# Supplementary material for: Screening of potential key ferroptosis-related genes in sepsis
Source: PeerJ. 2022 Sep 13;10:e13983. doi: 10.7717/peerj.13983 (PMC9480065; doi:10.7717/peerj.13983)
Supplement: Supplemental Information 10 [file peerj-10-13983-s010.pdf]

Category	Term	Count	%	Adj. p value	Gene ID
GOTERM_BP	stress-activated MAPK cascade	6	75	3.08E-07	MAPK14/DUSP1/MAP3K5/MAPK1/MAPK8/TLR4
GOTERM_BP	stress-activated protein kinase signaling cascade	6	75	3.08E-07	MAPK14/DUSP1/MAP3K5/MAPK1/MAPK8/TLR4
GOTERM_BP	response to oxidative stress	6	75	1.77E-06	CYBB/DUSP1/MAP3K5/MAPK1/MAPK8/TLR4
GOTERM_MF	protein serine/threonine kinase activity	5	62.5	1.16E-05	ATM/MAPK14/MAP3K5/MAPK1/MAPK8
GOTERM_BP	regulation of MAP kinase activity	5	62.5	1.76E-05	MAPK14/DUSP1/MAP3K5/MAPK1/TLR4
GOTERM_BP	cellular response to external stimulus	5	62.5	1.76E-05	CYBB/MAP3K5/MAPK1/MAPK8/TLR4
GOTERM_BP	activation of MAPK activity	4	50	4.24E-05	MAPK14/MAP3K5/MAPK1/TLR4
GOTERM_BP	cellular response to lipopolysaccharide	4	50	8.93E-05	MAPK14/MAPK1/MAPK8/TLR4
KEGG_PATHWAY	NOD-like receptor	5	62.5	2.26E-05	MAPK14/CYBB/MAPK1/MAPK8/TLR4

	signaling pathway				
KEGG_PATHWAY	Shigellosis	5	62.5	3.17E-05	ATM/MAPK14/MAPK1/MAPK8/TLR4
KEGG_PATHWAY	Toll-like receptor signaling pathway	4	50	3.17E-05	MAPK14/MAPK1/MAPK8/TLR4
KEGG_PATHWAY	TNF signaling pathway	4	50	3.41E-05	MAPK14/MAP3K5/MAPK1/MAPK8
KEGG_PATHWAY	MAPK signaling pathway	5	62.5	3.53E-05	MAPK14/DUSP1/MAP3K5/MAPK1/MAPK8
KEGG_PATHWAY	FoxO signaling pathway	4	50	4.56E-05	ATM/MAPK14/MAPK1/MAPK8
KEGG_PATHWAY	Apoptosis	4	50	4.76E-05	ATM/MAP3K5/MAPK1/MAPK8
KEGG_PATHWAY	Fluid shear stress and atherosclerosis	4	50	4.76E-05	MAPK14/DUSP1/MAP3K5/MAPK8
