# Supplementary material for: Screening of potential key ferroptosis-related genes in sepsis
Source: PeerJ. 2022 Sep 13;10:e13983. doi: 10.7717/peerj.13983 (PMC9480065; doi:10.7717/peerj.13983)
Supplement: Supplemental Information 11 [file peerj-10-13983-s011.pdf]

miRNAs	gene symbol	p. value
hsa-miR-940	ATM	6.16E-05
hsa-miR-92a-3p	ATM	2.83E-05
hsa-miR-8070	ATM	0.001092
hsa-miR-7160-5p	ATM	0.001327
hsa-miR-7151-3p	ATM	0.001556
hsa-miR-6893-5p	ATM	0.002737
hsa-miR-6884-5p	ATM	0.003374
hsa-miR-6808-5p	ATM	0.013218
hsa-miR-6771-5p	ATM	7.91E-05
hsa-miR-665	ATM	0.00081
hsa-miR-572	ATM	1.49E-06
hsa-miR-485-5p	ATM	2.34E-05
hsa-miR-4768-3p	ATM	0.00081
hsa-miR-4722-5p	ATM	1.72E-05
hsa-miR-4695-5p	ATM	0.000732
hsa-miR-4537	ATM	1.27E-06
hsa-miR-4478	ATM	0.001371
hsa-miR-3144-3p	ATM	2.41E-05
hsa-miR-2467-3p	ATM	0.002972

hsa-miR-223-3p	ATM	0.00403
hsa-miR-1910-3p	ATM	0.001371
hsa-miR-18a-3p	ATM	2.85E-05
hsa-miR-1827	ATM	0.0074
hsa-miR-7113-3p	CYBB	0.03071
hsa-miR-6892-3p	CYBB	7.25E-05
hsa-miR-6859-3p	CYBB	0.003539
hsa-miR-6734-3p	CYBB	0.003026
hsa-miR-4685-3p	CYBB	0.004018
hsa-miR-4640-3p	CYBB	0.014036
hsa-miR-34a-5p	CYBB	3.65E-05
hsa-miR-3183	CYBB	0.000124
hsa-miR-1282	CYBB	0.004039
hsa-miR-769-5p	MAPK1	2.75E-05
hsa-miR-766-3p	MAPK1	0.000428
hsa-miR-7111-3p	MAPK1	0.000184
hsa-miR-7110-3p	MAPK1	9.23E-05
hsa-miR-6873-3p	MAPK1	0.000209
hsa-miR-6867-3p	MAPK1	0.001951
hsa-miR-6862-5p	MAPK1	2.82E-05

hsa-miR-6857-5p	MAPK1	0.005105
hsa-miR-6853-5p	MAPK1	0.001112
hsa-miR-6846-3p	MAPK1	0.000352
hsa-miR-6831-5p	MAPK1	0.001132
hsa-miR-6817-3p	MAPK1	2.40E-05
hsa-miR-6813-5p	MAPK1	0.000153
hsa-miR-6810-5p	MAPK1	9.99E-05
hsa-miR-6808-3p	MAPK1	0.000138
hsa-miR-6777-3p	MAPK1	3.28E-07
hsa-miR-6763-5p	MAPK1	0.002692
hsa-miR-6741-3p	MAPK1	7.62E-07
hsa-miR-6734-3p	MAPK1	8.46E-08
hsa-miR-6720-5p	MAPK1	4.01E-08
hsa-miR-6512-3p	MAPK1	7.32E-05
hsa-miR-6499-3p	MAPK1	0.000214
hsa-miR-6085	MAPK1	0.002107
hsa-miR-585-3p	MAPK1	0.000214
hsa-miR-584-5p	MAPK1	2.15E-06
hsa-miR-5698	MAPK1	0.013437
hsa-miR-5581-5p	MAPK1	0.000239

hsa-miR-526b-3p	MAPK1	0.000114
hsa-miR-519d-3p	MAPK1	1.82E-06
hsa-miR-519b-3p	MAPK1	1.82E-06
hsa-miR-5190	MAPK1	0.000654
hsa-miR-504-3p	MAPK1	0.000153
hsa-miR-5000-5p	MAPK1	0.000926
hsa-miR-499a-3p	MAPK1	0.004295
hsa-miR-494-3p	MAPK1	0.00323
hsa-miR-487a-3p	MAPK1	0.001102
hsa-miR-4796-3p	MAPK1	0.009011
hsa-miR-4784	MAPK1	0.000617
hsa-miR-4770	MAPK1	0.000581
hsa-miR-4741	MAPK1	0.006498
hsa-miR-4725-3p	MAPK1	0.001825
hsa-miR-4708-5p	MAPK1	1.31E-05
hsa-miR-4692	MAPK1	0.000404
hsa-miR-4691-3p	MAPK1	5.17E-08
hsa-miR-4689	MAPK1	0.000566
hsa-miR-4675	MAPK1	0.000215
hsa-miR-4667-3p	MAPK1	0.000184

hsa-miR-4638-5p	MAPK1	8.89E-07
hsa-miR-4514	MAPK1	0.000569
hsa-miR-449b-3p	MAPK1	0.0014
hsa-miR-4433a-3p	MAPK1	7.39E-05
hsa-miR-4430	MAPK1	0.000166
hsa-miR-4420	MAPK1	0.005115
hsa-miR-4287	MAPK1	6.95E-06
hsa-miR-4271	MAPK1	0.005008
hsa-miR-4253	MAPK1	2.72E-05
hsa-miR-4252	MAPK1	0.000145
hsa-miR-3936	MAPK1	0.000638
hsa-miR-3927-5p	MAPK1	0.000161
hsa-miR-378a-3p	MAPK1	0.024706
hsa-miR-371b-3p	MAPK1	1.82E-06
hsa-miR-3666	MAPK1	0.005779
hsa-miR-3652	MAPK1	6.25E-06
hsa-miR-3609	MAPK1	0.001144
hsa-miR-3605-5p	MAPK1	0.006699
hsa-miR-329-3p	MAPK1	0.000249
hsa-miR-3150a-3p	MAPK1	1.03E-05

hsa-miR-3135b	MAPK1	7.24E-05
hsa-miR-28-5p	MAPK1	0.000629
hsa-miR-214-3p	MAPK1	2.85E-06
hsa-miR-2110	MAPK1	0.013437
hsa-miR-20b-5p	MAPK1	0.006513
hsa-miR-20a-5p	MAPK1	0.006513
hsa-miR-199a-3p	MAPK1	0.000131
hsa-miR-197-3p	MAPK1	7.62E-07
hsa-miR-196a-5p	MAPK1	0.002299
hsa-miR-18b-3p	MAPK1	0.003629
hsa-miR-181a-5p	MAPK1	0.000306
hsa-miR-17-5p	MAPK1	0.000676
hsa-miR-130b-3p	MAPK1	0.006987
hsa-miR-130a-3p	MAPK1	0.002241
hsa-miR-1255b-5p	MAPK1	0.0118
hsa-miR-1255a	MAPK1	0.0118
hsa-miR-122-5p	MAPK1	0.014047
hsa-miR-1200	MAPK1	3.46E-05
hsa-miR-106b-5p	MAPK1	0.000564
hsa-miR-106a-5p	MAPK1	0.000676

hsa-let-7b-5p	MAPK1	0.010863
hsa-miR-8055	MAPK14	0.000181
hsa-miR-7113-3p	MAPK14	0.011616
hsa-miR-6892-3p	MAPK14	0.002466
hsa-miR-6864-3p	MAPK14	0.000186
hsa-miR-6836-3p	MAPK14	0.000264
hsa-miR-6820-3p	MAPK14	0.000115
hsa-miR-6781-3p	MAPK14	3.79E-06
hsa-miR-6773-3p	MAPK14	0.000186
hsa-miR-6760-3p	MAPK14	0.008142
hsa-miR-6759-3p	MAPK14	2.82E-05
hsa-miR-6501-5p	MAPK14	8.69E-06
hsa-miR-643	MAPK14	0.000697
hsa-miR-513b-5p	MAPK14	5.85E-06
hsa-miR-4735-5p	MAPK14	0.004393
hsa-miR-4685-3p	MAPK14	0.011616
hsa-miR-4680-5p	MAPK14	0.000194
hsa-miR-4524a-3p	MAPK14	0.003198
hsa-miR-4469	MAPK14	0.019726
hsa-miR-4423-5p	MAPK14	0.010056

hsa-miR-4287	MAPK14	0.000256
hsa-miR-3672	MAPK14	0.001696
hsa-miR-2682-3p	MAPK14	0.001334
hsa-miR-24-3p	MAPK14	0.005943
hsa-miR-2276-5p	MAPK14	0.025896
hsa-miR-215-3p	MAPK14	3.63E-06
hsa-miR-214-3p	MAPK14	6.46E-06
hsa-miR-205-5p	MAPK14	0.011616
hsa-miR-199a-3p	MAPK14	0.00012
hsa-miR-186-3p	MAPK14	6.42E-05
hsa-miR-17-5p	MAPK14	7.45E-07
hsa-miR-155-5p	MAPK14	0.002215
hsa-miR-1267	MAPK14	0.001877
hsa-miR-125a-3p	MAPK14	0.001381
hsa-miR-1250-3p	MAPK14	0.00012
hsa-miR-124-3p	MAPK14	7.26E-06
hsa-miR-1234-3p	MAPK14	0.000939
hsa-miR-1208	MAPK14	0.002998
hsa-miR-1183	MAPK14	0.043633
hsa-miR-106a-5p	MAPK14	7.45E-07

hsa-miR-892b	MAPK8	0.000428
hsa-miR-8060	MAPK8	0.000924
hsa-miR-664a-3p	MAPK8	0.00802
hsa-miR-557	MAPK8	0.002092
hsa-miR-497-3p	MAPK8	0.001244
hsa-miR-496	MAPK8	0.000692
hsa-miR-4524b-5p	MAPK8	0.011921
hsa-miR-433-3p	MAPK8	0.006843
hsa-miR-3680-3p	MAPK8	5.25E-05
hsa-miR-214-3p	MAPK8	1.41E-07
hsa-miR-193b-3p	MAPK8	0.007551
hsa-miR-10a-5p	MAPK8	1.13E-06
hsa-miR-942-5p	TLR4	0.000188
hsa-miR-526b-5p	TLR4	0.000279
hsa-miR-4742-3p	TLR4	0.000444
hsa-miR-4738-3p	TLR4	0.000309
hsa-miR-4698	TLR4	0.003893
hsa-miR-4694-3p	TLR4	0.042042
hsa-miR-4659a-3p	TLR4	0.037545
hsa-let-7b-5p	TLR4	0.004797
