# Supplementary material for: Screening of potential key ferroptosis-related genes in sepsis
Source: PeerJ. 2022 Sep 13;10:e13983. doi: 10.7717/peerj.13983 (PMC9480065; doi:10.7717/peerj.13983)
Supplement: Supplemental Information 12 [file peerj-10-13983-s012.pdf]

miRNA	Target LncRNA	Pr. score
hsa-miR-199a-3p	MALAT1	0.724
	SNHG1	0.714
	HOXA10-HOXA9	0.706
	LINC00657	0.704
	NEAT1	0.614
	CASC7	0.571
	CTC-444N24.11	0.557
	BLOC1S5-TXNDC5	0.425
	ZFAS1	0.397
hsa-miR-17-5p	**CTB-89H12.4**	**0.922**
	**LINC00657**	**0.905**
	NEAT1	0.799
	MALAT1	0.732
	H19	0.56
	RNU12	0.527
	CROCCP2	0.416
hsa-miR-106a-5p	**CTB-89H12.4**	**0.996**
	**RP11-553L6.5**	**0.995**
	**NEAT1**	**0.935**

	**XLOC_013866**	**0.929**
	**RP1-309I22.2**	**0.911**
	MALAT1	0.797
	LINC00657	0.781
	RP11-217B1.2	0.607
	SCARNA10	0.496
	AC008746.12	0.382
hsa-let-7b-5p	**TRG-AS1**	**0.98**
	**TUG1**	**0.951**
	**NEAT1**	**0.907**
	**HOXA10-HOXA9**	**0.905**
	TTTY15	0.787
	XLOC_008295	0.787
	NUTM2A-AS1	0.672
	OIP5-AS1	0.534
	XLOC_013866	0.438
	VTRNA2-1	0.405
	H19	0.402
	RP11-227G15.3	0.4
	RP11-140H17.2	0.396

	LINC00910	0.393
	RPPH1	0.386
	XLOC_000992	0.386
	RNASEK-C17orf49	0.384
	MALAT1	0.371
	AC084082.3	0.367
hsa-miR-214-3p	**NEAT1**	**0.935**
	**C1RL-AS1**	**0.925**
	ABHD14A-ACY1	0.879
	CTD-3148I10.15	0.854
	AC005540.3	0.818
	CASC7	0.811
	IPO11-LRRC70	0.793
	PSMG3-AS1	0.735
	P2RX5-TAX1BP3	0.733
	SNHG16	0.726
	HEIH	0.692
	LINC00657	0.633
	AC005154.6	0.583
	SNHG12	0.574

MALAT1	0.551
RP1-178F10.3	0.528
OIP5-AS1	0.515
TMX2-CTNND1	0.5
BLOC1S5-TXNDC5	0.479
RP11-890B15.3	0.475
AC003092.1	0.465
MEG3	0.462
